# Efficacy and safety of glucagon-like peptide-1 receptor agonists in Parkinson’s disease: a systematic review and meta-analysis of randomized placebo-controlled clinical trials

**DOI:** 10.1177/17562864251408269

**Published:** 2026-01-31

**Authors:** Maria-Ioanna Stefanou, Evangelos Panagiotopoulos, Anastasios Tentolouris, Aikaterini Theodorou, Georgia Papagiannopoulou, Athanasia Athanasaki, Panagiota-Eleni Tsalouchidou, Melpomeni Peppa, Vaia Lambadiari, Spiridon Konitsiotis, Annerose Mengel, Georgios P. Paraskevas, Nikolaos Tentolouris, Georgios Tsivgoulis

**Affiliations:** Second Department of Neurology, “Attikon” University Hospital, School of Medicine, National and Kapodistrian University of Athens, Athens, Greece; Department of Neurology and Stroke, Eberhard-Karls University of Tübingen, Tübingen, Germany; Second Department of Neurology, “Attikon” University Hospital, School of Medicine, National and Kapodistrian University of Athens, Athens, Greece; First Department of Propaedeutic Internal Medicine and Diabetes Center, School of Medicine, National and Kapodistrian University of Athens, Laiko General Hospital, Athens, Greece; Second Department of Neurology, “Attikon” University Hospital, School of Medicine, National and Kapodistrian University of Athens, Athens, Greece; Second Department of Neurology, “Attikon” University Hospital, School of Medicine, National and Kapodistrian University of Athens, Athens, Greece; Second Department of Neurology, “Attikon” University Hospital, School of Medicine, National and Kapodistrian University of Athens, Athens, Greece; Second Department of Neurology, “Attikon” University Hospital, School of Medicine, National and Kapodistrian University of Athens, Athens, Greece; Diabetes Center, Second Department of Internal Medicine, “Attikon” University Hospital, Medical School, National and Kapodistrian University of Athens, Athens, Greece; Diabetes Center, Second Department of Internal Medicine, “Attikon” University Hospital, Medical School, National and Kapodistrian University of Athens, Athens, Greece; Department of Neurology, University Hospital of Ioannina, School of Health Sciences, University of Ioannina, Ioannina, Greece; Department of Neurology and Stroke, Eberhard-Karls University of Tübingen, Tübingen, Germany; Second Department of Neurology, “Attikon” University Hospital, School of Medicine, National and Kapodistrian University of Athens, Athens, Greece; First Department of Propaedeutic Internal Medicine and Diabetes Center, School of Medicine, National and Kapodistrian University of Athens, Laiko General Hospital, Athens, Greece; Second Department of Neurology, “Attikon” University Hospital, School of Medicine, National and Kapodistrian University of Athens, Rimini 1, Chaidari, Athens 12462, Greece

**Keywords:** exenatide, GLP-1, GLP-1 receptor agonist, lixisenatide, Parkinson’s disease

## Abstract

**Background:**

Converging lines of preclinical evidence support the neuroprotective properties of glucagon-like peptide-1 receptor agonists (GLP-1 RAs) in Parkinson’s disease (PD). Nevertheless, results from randomized-controlled clinical trials (RCTs) remain conflicting.

**Objectives:**

To assess the safety and efficacy of GLP-1 RAs in PD.

**Design:**

Systematic review and meta-analysis of randomized placebo-controlled clinical trials.

**Data sources and methods:**

A systematic search of MEDLINE and Scopus databases was conducted on October 7, 2025, for randomized placebo-controlled clinical trials investigating GLP-1 RAs in adults with PD. Risk of bias was evaluated using the Cochrane Collaboration risk-of-bias (RoB2) tool.

**Results:**

Four RCTs comprising 667 PD patients (377 receiving GLP-1 RAs) were included. Between baseline and end-of-treatment, no differences were observed in the Movement Disorder Society-Unified Parkinson’s Disease Rating Scale (MDS-UPDRS) Part III score change between GLP-1 RA- and placebo-treated patients in either off-medication (standardized mean difference (SMD): −0.16; 95% CI: −0.64 to 0.32; *p* = 0.52) or on-medication states (SMD: −0.13; 95% confidence interval (CI): −0.51 to 0.25; *p* = 0.49). No significant differences were uncovered in other MDS-UPDRS subscores, Non-Motor Symptoms Scale, Montreal Cognitive Assessment, or Parkinson’s Disease Questionnaire scores. The risk of serious adverse events and odds of treatment discontinuation were similar between groups, but GLP-1 RAs were associated with an increased risk of weight loss compared to placebo (risk ratio: 1.44; 95% CI: 1.04–1.99; *p* = 0.03).

**Conclusion:**

GLP-1 RAs were not associated with improvements in motor or non-motor domains of PD. However, robust preclinical evidence and promising findings in select subpopulations warrant further RCTs to evaluate their neuroprotective potential, prioritizing long-acting and brain-penetrant agents that effectively engage central GLP-1 circuits for PD treatment.

**Registration:**

The pre-specified protocol of the present systematic review and meta-analysis has been registered in the International Prospective Register of Ongoing Systematic Reviews PROSPERO (registration ID: CRD420251008703).

## Plain language summary

Efficacy and safety of glucagon-like peptide-1 receptor agonists in Parkinson’s disease: a systematic review and meta-analysis

Glucagon-like peptide-1 receptor agonists (GLP-1 RAs) are medications approved for type 2 diabetes and obesity that have shown neuroprotective effects in preclinical models of Parkinson’s disease (PD). However, clinical trial results have been inconsistent. In this systematic review and meta-analysis, we evaluated the overall safety and effectiveness of GLP-1 RAs in people with PD by analyzing data from all available randomized placebo-controlled clinical trials. Our findings indicate that GLP-1 RAs do not lead to measurable improvements in motor symptoms, cognitive performance, or quality of life when compared to placebo. Although the risk of serious adverse events was similar between treatment and placebo groups, GLP-1 RAs were associated with an increased risk of weight loss and gastrointestinal adverse events. Despite the lack of demonstrated clinical benefit, strong preclinical evidence and exploratory signals in specific patient subgroups support the need for further high-quality trials, particularly with long-acting and brain-penetrant GLP-1 RAs in early-stage PD populations.

## Introduction

Parkinson’s disease (PD) is the second most common neurodegenerative disorder and the neurological disease with the fastest rising prevalence and disability worldwide.^1^ Large-scale epidemiological studies forecast that by 2050, the global number of PD cases will reach 25.2 million, with population aging, environmental pollution, and the toxic milieu associated with urbanization and industrialization poised to comprise the main determinants of the incremental rise in PD prevalence, projected to reach 267 cases per 100,000 population by 2050.^2^ As the current mainstay of PD management mainly relies on symptomatic treatments, there is a pressing healthcare demand for disease-modifying and neuroprotective therapies that may forestall disease progression and functional disability.^3^

In this regard, there is converging evidence suggesting that the repurposing of glucagon-like peptide-1 receptor agonists (GLP-1 RAs)—currently licensed for the treatment of type 2 diabetes mellitus (T2DM) and obesity—could hold promise for PD treatment.^4^ First, epidemiological studies have previously disclosed an inverse association between GLP-1 RA use and the risk of PD in patients with T2DM, with a population-based cohort of more than 100,000 T2DM patients demonstrating a 62% reduction in the risk of developing PD in GLP-1 RA-treated patients compared to controls receiving other antidiabetic agents.^4^ Second, given the central role of the gut–brain axis in PD pathology, GLP-1 signaling represents a highly attractive target for PD treatment. GLP-1 RAs exert peripheral neuroprotective effects by binding to vagal afferent neurons, which are also implicated in α-synuclein propagation in PD.^5–7^ In addition, GLP-1 RAs can readily cross the blood–brain barrier to exert central effects by binding to GLP-1 receptors in the brain, which are abundantly expressed in subcortical and cortical networks implicated in PD.^8,9^ Third, preclinical research in animal models has provided compelling evidence that GLP-1 RAs harbor strong neuroprotective potential, dampening neuroinflammation, including microglial activation, exerting anti-apoptotic effects on nigrostriatal dopaminergic neurons, and improving motor performance in rodent models of PD.^10–12^

During the past few years, proof-of-concept clinical trials have emerged,^13^ providing preliminary translational evidence of clinically relevant improvements in motor and cognitive performance in PD patients treated with GLP-1 RAs, followed by larger randomized-controlled clinical trials (RCTs) with incongruent findings.^3,14^ A 2020 Cochrane meta-analysis on GLP-1 RAs in PD,^15^ which included one double-blind, phase II RCT in patients with PD receiving the GLP-1 RA exenatide versus placebo^16^ and one single-blind, phase II RCT in PD patients receiving exenatide versus no treatment,^13^ concluded that exenatide may improve motor impairment in PD. However, the limited number of RCTs and the very low certainty of evidence cautioned against drawing unequivocal conclusions without further validation of these data. Since 2020, additional RCTs on GLP-1 RAs in PD have been published, while mounting evidence on the pleiotropic neuroprotective properties of GLP-1 RAs has stirred interest regarding their potential utility in neurodegenerative disorders, extending beyond PD to include Alzheimer’s disease and vascular dementia.^15,17,18^

The aim of the present systematic review and meta-analysis is to provide a comprehensive and updated assessment of the safety and efficacy of GLP-1 RAs in PD, incorporating data from all randomized placebo-controlled clinical trials conducted to date in PD patient populations.

## Methods

### Standard protocol approvals and registrations

Reporting complies with the Preferred Reporting Items for Systematic Reviews and Meta-Analyses (PRISMA) statement.^19^ In accordance with the study design (systematic review and meta-analysis), no Ethical Committee approval was required. The study protocol, encompassing a predefined PICOS (Population, Intervention, Comparison, Outcome, and Study) framework, was a priori designed and registered in the PROSPERO database (CRD420251008703). All supporting data are available within the article and its Supplemental Files.

### Data sources and searches

Three independent reviewers (M.-I.S., E.P., An.T.) searched for published randomized placebo-controlled clinical trials testing GLP-1 RAs in adults with PD. Eligible studies were identified by a systematic search in MEDLINE (via PubMed) and Scopus databases. The combination of search strings for all database queries included combined search terms: “glucagon-like peptide-1 receptor agonist,” “exenatide,” “lixisenatide,” “semaglutide,” “albiglutide,” “liraglutide,” “dulaglutide,” and “Parkinson’s disease.” The full search algorithms used in MEDLINE and Scopus databases are provided in the Supplemental Material. Our search was restricted to randomized placebo-controlled trials, while no language restrictions were applied. The search spanned from each electronic database’s inception to October 7, 2025. Manual screening of the bibliographies of articles meeting the study’s inclusion criteria was additionally conducted to enhance the comprehensiveness of the literature.^20,21^

Randomized placebo-controlled clinical trials in adults with PD under treatment with GLP-1 RAs were eligible for inclusion. Exclusion criteria comprised: (1) preclinical studies; (2) studies not including ascertained PD cases based on current diagnostic criteria^22^; (3) studies reporting outcomes that did not align with the predefined inclusion criteria; and (4) narrative and systematic reviews, case-series or case-reports, commentaries, preprints or non-peer reviewed studies, and conference abstracts. In case of studies with overlapping data, the study with the largest dataset was retained. All retrieved studies were independently assessed by three reviewers (M.-I.S., E.P., An.T.), and disagreements were resolved by consensus after discussion with a fourth tie-breaking evaluator (G.T.).

### Quality control, bias assessment, and data extraction

For relevant domains of each included study, the risk of bias was assessed using the Cochrane Collaboration risk-of-bias (RoB2) tool for RCTs.^23^ Three independent reviewers (M.-I.S., E.P., An.T.) performed quality control and bias assessment, and in case of disagreement, consensus after discussion with the corresponding author (G.T.) was reached. Data, including first author name, publication year, study design and duration, patient population, sample size, and outcomes, were extracted from individual studies in structured reports.^24^

### Outcomes

An aggregate data meta-analysis was performed, including all identified studies. The primary efficacy outcome was the change in Movement Disorder Society-Unified Parkinson’s Disease Rating Scale (MDS-UPDRS) part III at “OFF” and “ON” medication state. Secondary efficacy outcomes comprised changes in (i) MDS-UPDRS part I, (ii) MDS-UPDRS part II, (iii) MDS-UPDRS part IV, (iv) Non-Motor Symptoms Scale (NMSS) at “ON” state, (v) Montreal Cognitive Assessment (MoCA) at “ON” state, and (vi) the 39-item Parkinson’s Disease Questionnaire (PDQ-39) at “ON” state. All primary and secondary efficacy outcomes of interest were assessed at the end of the treatment period. To enable comparability and data pooling across trials with or without wash-out periods, all efficacy outcomes were assessed at the end of the active treatment phase, as reported in each included study. The primary safety outcomes were (i) the pooled risk ratio (RR) of serious adverse events (SAEs) in patients treated with GLP-1 RAs compared to those receiving placebo, and (ii) the pooled RR of SAEs and adverse events (AEs) leading to treatment discontinuation in GLP-1 RA-treated versus placebo-treated patients. Secondary safety outcomes comprised the pooled RR of weight loss (defined as the change in total body weight from baseline) among patients with PD treated with GLP-1 RAs compared to those receiving placebo.

### Statistical analysis

R-software version 3.5.0 (packages: meta and metafor) was used for meta-analysis. Continuous outcomes measured in dissimilar ways were analyzed using standardized mean difference (SMD) estimates, calculated as the mean differences (MDs) divided by the corresponding pooled standard deviations and were subsequently interpreted using estimates proposed by Cohen, according to which an SMD of 0.2 represents a small effect, an SMD of 0.5 represents a medium effect, and an SMD of ⩾0.8 represents a large effect.^25^ In the cases that no events were observed for assessed outcomes in included studies, a continuity correction was performed in accordance with the Cochrane Handbook. For sensitivity analyses, continuous outcomes were assessed using MD and their corresponding 95% confidence intervals (95% CI). For dichotomous outcomes of interest, the pooled RR with its corresponding 95% CI was calculated.^26^ All estimates were pooled under the random-effects model.^27^ Heterogeneity was assessed with the *I*^2^ and Cochran *Q* statistics. For the qualitative interpretation of heterogeneity, *I*^2^ values >50% and values >75% were considered to represent substantial and considerable heterogeneity, respectively. The significance level was set at 0.1 for the *Q* statistic,^28^ while the equivalent *z* test with a two-tailed *p*-value < 0.05 was considered statistically significant for each pooled estimate.

### Data availability statement

All data generated or analyzed during this study are included in this article and its Supplemental Information Files.

## Results

### Literature search and included studies

The systematic database search yielded a total of 26 and 70 records from the MEDLINE and Scopus databases, respectively (Figure S1). After excluding duplicates and initial screening, we retrieved the full text of eight records that were considered potentially eligible for inclusion. After reading the full-text articles, four were further excluded (Table S1). Finally, we identified 4 eligible studies for inclusion in the systematic review and meta-analysis,^3,14,16,29^ comprising a total of 667 patients with PD, receiving either GLP-1 RA treatment (*n* = 377) or placebo (*n* = 290; Table 1). Notably, for the NCT04154072 trial, in which patients were randomly allocated with a 1:1:1 ration to one of two active treatment groups (2.5 or 5 mg NLY01) or matching placebo, since extrapolation or synthesis of data was otherwise not possible, only the subgroup that received the high dose of NLY01 (5 mg; *n* = 85) was included in the subsequent analyses of primary and secondary outcomes.^3^

**Table 1.** Main characteristics of studies included in the meta-analysis.

Study	Year	Medication/type	Study design/country	Population	Study period	Total population (*n*)	Active (*n*)	Placebo (*n*)
Athauda et al.^16^	2017	Exenatide 2 mg once weekly or placebo SC/GLP-1 RA	Single-center, randomized, parallel-arm, double-blind, placebo-controlled trial/UK	Patients with moderate PD, on dopaminergic treatment, Hoehn & Yahr stage ⩽2.5	48 weeks + 12-week washout	62	32	30
McGarry et al.^3^	2024	NLY01 (2.5 or 5.0 mg) SC weekly or placebo/GLP-1 RA	Multicenter, randomized, double-blind, placebo-controlled trial/USA	Early untreated PD consistent with UK Brain Bank or MDS criteria, DaT imaging consistent with PD, age 30–80, Hoehn & Yahr ⩽2.5, MoCA ⩾24	36 weeks	255	170 (85 in high dose, 85 in low dose)	85
Meissner et al.^14^	2024	Lixisenatide 10 µg daily for 14 days, then 20 µg daily SC or placebo/GLP-1 RA	Multicenter, randomized, double-blind, placebo-controlled trial/France	PD diagnosed <3 years, age 40–75, Hoehn & Yahr <3, stable dopaminergic therapy ⩾1 month, MoCA ⩾26	12 months + 2-month washout	156	78	78
Vijiaratnam et al.^29^	2025	Exenatide 2 mg once weekly SC or placebo/GLP-1 RA	Multicenter, randomized, double-blind, placebo-controlled trial/UK	PD, Hoehn & Yahr stage ⩽2.5, on stable dopaminergic treatment	96 weeks	194	97	97

DaT, dopamine transporter; GLP-1 RA, glucagon-like peptide-1 receptor agonist; MDS, Movement Disorder Society; MoCA, Montreal Cognitive Assessment; PD, Parkinson’s disease; SC, subcutaneous.

### Quality control and publication bias of included studies

The risk-of-bias assessment of included studies using the Cochrane RoB2 tool^23^ is presented in Figures S2 and S3. All four RCTs demonstrated robust methodology with a low risk of bias in key domains, including randomization, allocation concealment, adherence to the intervention, handling of missing data, and selection of the reported results. However, each trial was rated as having some concerns due to potential limitations in the measurement of certain outcomes, particularly those relying on patient-reported assessments.^3,14,16,29^

All funnel plots assessing publication bias among trials reporting the outcomes are compiled in the Supplemental Material (Figures S4, S5, S7, S9, S11, S13, S15, S17, S20, and S21).

### Quantitative analyses

**Figure 1.**
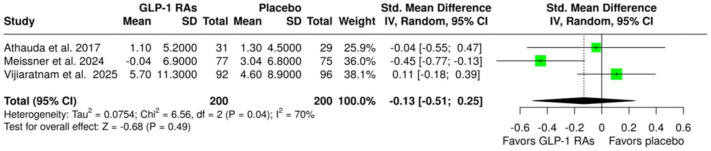
Forest plot comparing the changes in MDS-UPDRS Part III scores at “OFF” state in patients with Parkinson’s disease treated with GLP-1 RAs versus placebo. GLP-1 RAs, glucagon-like peptide-1 receptor agonists; MDS, UPDRS, Movement Disorder Society-Unified Parkinson’s Disease Rating Scale.

**Figure 2.**
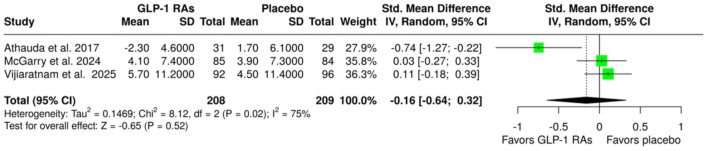
Forest plot comparing the changes in MDS-UPDRS Part III scores at “ON” state in patients with Parkinson’s disease treated with GLP-1 RAs versus placebo. GLP-1 RAs, glucagon-like peptide-1 receptor agonists; MDS, UPDRS, Movement Disorder Society-Unified Parkinson’s Disease Rating Scale.

An overview of the analyses for primary and secondary outcomes is summarized in Table 2. With respect to the primary efficacy outcomes, no association was observed between treatment with GLP-1 RAs and changes in MDS-UPDRS Part III scores at either the “OFF” state (SMD: −0.16; 95% CI: −0.64 to 0.32; *p* = 0.52; three studies; *I*^2^ = 75%; *p* for Cochran’s *Q* = 0.02; Figure 1) or the “ON” state (SMD: −0.13; 95% CI: −0.51 to 0.25; *p* = 0.49; three studies; *I*^2^ = 70%; *p* for Cochran’s *Q* = 0.04; Figure 2) at the end of the treatment period. However, the analysis revealed significant heterogeneity, indicating considerable variability among the included studies.

**Table 2.** Overview of the primary and secondary outcomes in patients with Parkinson’s disease treated with GLP-1 RAs versus placebo.

Clinical outcome	Statistical measure	Pooled outcome	*p*-Value	Heterogeneity (*I*^2^, *p* for Cochran *Q*)
MDS-UPDRS Part III (“OFF” state)	SMD, 95% CI	−0.16 (−0.64 to 0.32)	0.52	75%, 0.02
MDS-UPDRS Part III (“ON” state)	SMD, 95% CI	−0.13 (−0.51 to 0.25)	0.49	70%, 0.04
MDS-UPDRS Part I	SMD, 95% CI	0.03 (−0.15 to 0.20)	0.76	8%, 0.35
MDS-UPDRS Part II	SMD, 95% CI	0.04 (−0.13 to 0.20)	0.64	0%, 0.72
MDS-UPDRS Part IV	SMD, 95% CI	−0.03 (−0.23 to 0.17)	0.77	0%, 0.95
Non-Motor Symptoms Severity Scale (“ON” state)	SMD, 95% CI	−0.01 (−0.21 to 0.18)	0.89	0%, 0.81
MoCA (“ON” state)	SMD, 95% CI	0.05 (−0.15 to 0.24)	0.62	20%, 0.28
Parkinson’s Disease Questionnaire 39 (“ON” state)	SMD, 95% CI	−0.03 (−0.20 to 0.13)	0.71	0%, 0.96
Risk of SAEs	RR, 95% CI	0.97 (0.14–6.72)	0.97	0%, 0.97
Risk of SAEs or AEs leading to treatment discontinuation	RR, 95% CI	2.19 (0.39–12.38)	0.37	59%, 0.06
Risk of weight loss	RR, 95% CI	1.44 (1.04–1.99)	0.03	15%, 0.32

AEs, adverse events; CI, confidence interval; GLP-1 RAs, glucagon-like peptide-1 receptor agonists; MDS-UPDRS, Movement Disorder Society-Unified Parkinson’s Disease Rating Scale; MoCA, Montreal Cognitive Assessment; RR, risk ratio; SAEs, serious adverse events; SMD, standardized mean difference.

Regarding secondary efficacy outcomes, no significant associations were observed between treatment with GLP-1 RAs and changes in MDS-UPDRS Part I (SMD: 0.03; 95% CI: −0.15 to 0.20; *p* = 0.76; four studies; *I*^2^ = 8%; *p* for Cochran’s *Q* = 0.35; Figure S6), MDS-UPDRS Part II (SMD: 0.04; 95% CI: −0.13 to 0.20; *p* = 0.64; four studies; *I*^2^ = 0%; *p* for Cochran’s *Q* = 0.72; Figure S8), or MDS-UPDRS Part IV (SMD: −0.03; 95% CI: −0.23 to 0.17; *p* = 0.77; three studies; *I*^2^ = 0%; *p* for Cochran’s *Q* = 0.95; Figure S10) at the end of the treatment period. Similarly, no significant associations were observed between GLP-1 RA treatment and changes in the Non-Motor Symptoms Severity Scale at “ON” state (SMD: −0.01; 95% CI: −0.21 to 0.18; *p* = 0.89; three studies; *I*^2^ = 0%; *p* for Cochran’s *Q* = 0.81; Figure S12), the MoCA score at “ON” state (SMD: 0.05; 95% CI: −0.15 to 0.24; *p* = 0.62; three studies; *I*^2^ = 20%; *p* for Cochran’s *Q* = 0.28; Figure S14), or the PDQ-39 at “ON” state (SMD: −0.03; 95% CI: −0.20 to 0.13; *p* = 0.71; four studies; *I*^2^ = 0%; *p* for Cochran’s *Q* = 0.96; Figure S16) at the end of the treatment period.

**Figure 3.**
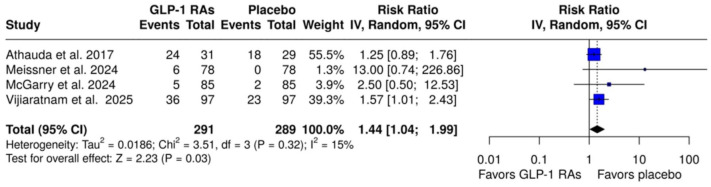
Forest plot comparing the risk of weight loss in patients with Parkinson’s disease treated with GLP-1 RAs versus placebo. GLP-1 RAs, glucagon-like peptide-1 receptor agonists.

With respect to safety outcomes, there was no significant difference in the risk of SAEs between patients treated with GLP-1 RAs and those receiving placebo (RR: 0.97; 95% CI: 0.14–6.72; *p* = 0.97; two studies; *I*^2^ = 0%; *p* for Cochran’s *Q* = 0.97; Figure S18). In addition, the pooled analysis did not demonstrate significant associations between GLP-1 RA treatment and the risk of SAEs or AEs that led to treatment discontinuation (RR: 2.19; 95% CI: 0.39–12.38; *p* = 0.37; four studies; *I*^2^ = 59%; *p* for Cochran’s *Q* = 0.06; Figure S19). However, substantial heterogeneity (*I*^2^ = 59%) was observed, suggesting variability across studies that may have influenced the pooled effect measure. A statistically significant association was observed between GLP-1 RAs and the risk of weight loss compared to placebo (RR: 1.44; 95% CI: 1.04–1.99; *p* = 0.03; four studies; *I*^2^ = 15%; *p* for Cochran’s *Q* = 0.32; Figure 3). In addition, the risk of gastrointestinal AEs, including nausea (RR: 2.61; 95% CI: 1.83–3.72; *p* < 0.01; four studies; *I*^2^ = 42%; *p* for Cochran’s *Q* = 0.15; Figure S22) and vomiting (RR: 5.01; 95% CI: 2.14–11.74; *p* < 0.01; four studies; *I*^2^ = 38%; *p* for Cochran’s *Q* = 0.38; Figure S23) was significantly increased with GLP-1 RA treatment, while the risks of diarrhea, constipation, and abdominal pain were comparable between groups (Figures S24–S26).

Sensitivity analyses did not affect any of the primary or secondary efficacy outcomes, and no significant differences were uncovered between GLP-1 RA- versus placebo-treated patients (Figures S27 and S34).

## Discussion

In the present systematic review and meta-analysis, we found no therapeutic benefit from GLP-1 RA treatment in patients with PD, as evidenced by the absence of significant differences in any measure of PD severity, including both motor and non-motor domains, between GLP-1 RA-treated and placebo-treated patients. Notably, the present evidence contrasts with prior positive RCT findings—particularly regarding motor performance—from two previous phase II trials: one on exenatide (NCT01971242)^16^ and one on lixisenatide (NCT03439943, the LIXIPARK trial).^14^ Specifically, regarding the primary efficacy outcomes, the pooled estimates for the MDS-UPDRS Part III scores in both OFF- and ON-medication states were primarily influenced by the recently published phase III RCT (NCT04232969),^29^ the largest and longest trial on GLP-1 RAs in PD to date. This trial, which included 194 patients with PD at Hoehn and Yahr stage 2.5 or less while on dopaminergic treatment and had an overall duration of 96 weeks, failed to demonstrate any disease-modifying effects of 2 mg extended-release exenatide administered once weekly compared to placebo.

It should be emphasized, however, that significant heterogeneity was uncovered in the pooled analyses of the primary efficacy outcomes. Disparities in patient characteristics and PD populations (i.e., early vs moderate PD stages, with vs without concomitant dopaminergic therapy), along with differences in GLP-1 RA (short- vs long-acting), in RCT design (i.e., trial duration, single- vs multicentric settings, sample size, and definitions of primary endpoints in OFF- or ON-medication states) may account for the discordant findings across trials. Importantly, two of the four included RCTs tested the short-acting GLP-1 RA exenatide, while one RCT assessed NLY01, a pegylated, brain-penetrant, longer-lasting version of exenatide (with a half-life of approximately 12.5 days).^3,16,29^ Notably, we identified no additional RCTs in PD evaluating long-acting GLP-1 receptor agonists (i.e., liraglutide, dulaglutide, semaglutide), which provide superior cardio-renal benefits over short-acting agents and could hold greater therapeutic potential.^20,21^ LIXIPARK^14^ was the only RCT to evaluate lixisenatide, a GLP-1 RA with nearly fourfold and threefold higher affinity for the GLP-1 receptor compared to native human GLP-1 and exenatide (the synthetic compound derived from the reptilian hormone exendin-4), respectively.^30,31^ Beyond signaling potency, discrepancies in GLP-1 receptor trafficking through non-GLP-1 receptor-related pathways, along with differences in blood–brain barrier permeability and central nervous system (CNS) bioavailability, suggest that lixisenatide may exhibit different neuroprotective properties compared with exenatide.^32^ Aligned with this hypothesis, a study using the 1-methyl-4-phenyl-1,2,3,6-tetrahydropyridine (MPTP) mouse model of PD demonstrated that lixisenatide, but not exendin-4, prevented apoptosis of dopaminergic neurons and MPTP-induced motor impairment.^11^ However, well-designed, longer phase III trials of lixisenatide in PD are warranted to determine whether these preclinical findings translate into clinical benefit, expanding the results of the LIXIPARK trial.^14^

Regarding secondary outcomes, our meta-analysis found no evidence of clinical improvement in motor and non-motor features of PD, including both patient- and clinician-reported outcome measures (MDS-UPDRS parts I, II, and IV; NMSS, MoCA, and PDQ-39). Accordingly, the lack of measurable effects from GLP-1 RAs in motor and non-motor domains was reflected in the striatal tracer binding patterns, which showed no significant differences between GLP-1 RA- and placebo-treated PD patients in RCTs that included DaT-SPECT imaging outcomes.^16,29^ Although a line of preclinical evidence suggests that GLP-1 circuits in the CNS can be engaged via peripheral administration of GLP-1 RAs, it is noteworthy that only 1% of the circulating plasma exenatide was detectable in the cerebrospinal fluid of treated PD patients.^29,33^ Assuming that efficient GLP-1 receptor signaling is a prerequisite for central neuromodulation, optimized GLP-1 RA dosing, improved CNS penetration, and optimal target engagement remain critical issues that warrant further exploration in PD trials.^34^

Some additional key aspects of the present findings should be elaborated. First, despite the well-established implication of GLP-1 in gastrointestinal motility and absorption, there are currently no data regarding the potential effects of GLP-1 RAs on the pharmacokinetic and pharmacodynamic properties of dopaminergic drugs in PD. Given that the effects of GLP-1 RAs on motor outcomes may be confounded by fluctuations in dopaminergic drug levels—particularly in RCTs where the MDS-UPDRS Part III in the ON-medication state serves as the primary endpoint (e.g., the LIXIPARK trial)—it is crucial to distinguish between symptomatic and disease-modifying effects using robust trial designs that additionally account for variations in dopaminergic treatment levels. Second, in line with preclinical evidence supporting the neuroprotective potential of GLP-1 RAs, subgroup analyses from two RCTs in PD indicated the possibility of clinically relevant motor benefits in younger (<60 years) patients or those at the earliest disease stages.^3,14^ Thus, future trials should prioritize the inclusion of patients at early or prodromal disease stages to investigate whether GLP-1 RAs can readily halt disease progression or prevent the onset of PD.

Regarding safety outcomes, there was no significant difference in the risk of SAEs or in the risk of SAEs or AEs leading to treatment discontinuation between GLP-1 RA- and placebo-treated patients. However, gastrointestinal AEs were frequent, with a significantly higher risk of nausea and vomiting associated with active GLP-1 RA treatment. In addition, GLP-1 RAs were associated with a significant increase in the risk of weight loss, a finding that warrants caution regarding the clinical generalizability of GLP-1 RAs in the PD patient population—especially considering that body-mass index (BMI) criteria were used to exclude underweight patients from the present trials. This limitation highlights the need for real-world studies to evaluate the safety of GLP-1 RAs in broader PD patient populations. The safety and tolerability of GLP-1 RAs in relation to weight loss will likely constitute a critical issue for clinical practice, given the association between frailty and sarcopenia with morbidity, risk of falls, and mortality in PD.^29,35^ Notably, further safety evidence is awaited from ongoing RCTs investigating GLP-1 RAs with more potent weight-loss-inducing effects, such as semaglutide (NCT03659682).

Besides the acknowledged sources of heterogeneity revealed in the pooled analyses of the primary outcomes, some additional limitations of the present meta-analysis should be outlined. First, only four RCTs were included, and the statistical power of this meta-analysis is restricted by both the limited number of studies and their small sample sizes, as well as the imprecision of several effect estimates with wide CIs. Consequently, the analysis may be underpowered and at risk of type II error. Notably, only one was a phase III trial. Despite the negative findings of this trial and based on compelling preclinical evidence and indications of benefit from GLP-1 RAs in selected patient subgroups, larger, well-designed RCTs focusing on CNS-penetrant GLP-1 RAs are warranted to explore their potential symptomatic or disease-modifying role in PD.^36^ Second, given the limited number of included studies, visual inspection of funnel-plot symmetry is inherently unreliable, and the sample size does not meet the minimum requirements for statistical assessments of publication bias, including Egger’s regression test. Because a robust evaluation of publication bias was not possible, this represents an important limitation of the available evidence. Third, given that GLP-1 RAs induce potent weight-loss effects but also frequently gastrointestinal AEs, it has been suggested that GLP-1 RAs may be associated with unmasking bias in clinical trials. Therefore, the inclusion of robust and objective neurophysiological outcomes (e.g., wearable sensors or monitoring devices, such as Parkinson’s Holter) or neuroimaging outcomes (e.g., DaTscan) should complement clinical RCT endpoints.^37^ Fourth, it is important to note that patients with concomitant T2DM have been excluded from the PD trials on GLP-1 RAs conducted thus far. Nonetheless, there is growing recognition that insulin resistance is a key determinant in the pathogenesis of PD,^38^ contributing to neuroinflammation, mitochondrial dysfunction, dopaminergic neuronal apoptosis, autophagy, and α-synuclein aggregation, and that T2DM is a predisposing risk factor for developing PD.^39^ Therefore, targeted assessment of GLP-1 RAs in subpopulations with PD and concomitant T2DM or prediabetes would be a clinically relevant avenue for future research. Fifth, a meta-analysis comparing the magnitude of weight loss between treatment groups, or assessing outcomes by body-weight category, was not feasible owing to inconsistent reporting across trials (i.e., lack of BMI stratification). In one RCT evaluating exenatide over a 48-week treatment period,^16^ participants in the active treatment arm experienced a mean weight loss of 2.6 kg, compared to 0.6 kg in the control group, yielding a between-group difference of 2.0 kg. In contrast, the most recent RCT investigating exenatide over a 96-week period reported an average reduction of 1.8 kg in the exenatide group versus 1.3 kg in the placebo group, corresponding to a difference of 0.5 kg.^29^ Given the signal of an increased risk of weight loss observed in the current meta-analysis, granular monitoring and reporting of body weight, fat mass, and lean mass should be considered in future studies.^40^ In addition, sensitivity analyses to assess the potential impact of weight loss-related effects on primary outcomes should be incorporated into future trial designs.

The main strength of the present meta-analysis lies in the robust meta-analytical framework employed: in accordance with the predefined protocol, only strictly placebo-controlled RCTs were included, while non-placebo-controlled trials, preprints, and conference abstracts were excluded. The rigorous selection criteria account for inconsistencies between the present study’s findings and prior meta-analyses investigating GLP-1 RAs for PD,^41–45^ which reported promising effects largely owing to methodological issues related to inclusion of non-placebo-controlled proof-of-concept trials,^13^ non-peer-reviewed preprints,^46^ and conference abstracts.^47^ Thus, the present meta-analysis of placebo-controlled RCTs at low risk of bias provides solid meta-analytic evidence on GLP-1 RAs use in PD.

## Conclusion

In conclusion, we found no evidence of symptomatic or disease-modifying effects of GLP-1 RAs on motor or non-motor features of PD compared to placebo. Nonetheless, preclinical evidence and promising findings in specific PD subpopulations advocate for further well-designed RCTs to evaluate the neuroprotective potential of optimized and longer-acting agents with enhanced blood–brain barrier penetration and improved GLP-1 receptor engagement in CNS networks implicated in PD pathogenesis. The prevalence and extent of weight loss should be included as an important safety consideration in future RCTs.

## Supplemental Material

sj-docx-1-tan-10.1177_17562864251408269 – Supplemental material for Efficacy and safety of glucagon-like peptide-1 receptor agonists in Parkinson’s disease: a systematic review and meta-analysis of randomized placebo-controlled clinical trials Supplemental material, sj-docx-1-tan-10.1177_17562864251408269 for Efficacy and safety of glucagon-like peptide-1 receptor agonists in Parkinson’s disease: a systematic review and meta-analysis of randomized placebo-controlled clinical trials by Maria-Ioanna Stefanou, Evangelos Panagiotopoulos, Anastasios Tentolouris, Aikaterini Theodorou, Georgia Papagiannopoulou, Athanasia Athanasaki, Panagiota-Eleni Tsalouchidou, Melpomeni Peppa, Vaia Lambadiari, Spiridon Konitsiotis, Annerose Mengel, Georgios P. Paraskevas, Nikolaos Tentolouris and Georgios Tsivgoulis in Therapeutic Advances in Neurological Disorders

sj-pdf-2-tan-10.1177_17562864251408269 – Supplemental material for Efficacy and safety of glucagon-like peptide-1 receptor agonists in Parkinson’s disease: a systematic review and meta-analysis of randomized placebo-controlled clinical trials Supplemental material, sj-pdf-2-tan-10.1177_17562864251408269 for Efficacy and safety of glucagon-like peptide-1 receptor agonists in Parkinson’s disease: a systematic review and meta-analysis of randomized placebo-controlled clinical trials by Maria-Ioanna Stefanou, Evangelos Panagiotopoulos, Anastasios Tentolouris, Aikaterini Theodorou, Georgia Papagiannopoulou, Athanasia Athanasaki, Panagiota-Eleni Tsalouchidou, Melpomeni Peppa, Vaia Lambadiari, Spiridon Konitsiotis, Annerose Mengel, Georgios P. Paraskevas, Nikolaos Tentolouris and Georgios Tsivgoulis in Therapeutic Advances in Neurological Disorders

## Declarations

**Ethics approval and consent to participate:** This study did not require an ethical board approval or written informed consent by the patients according to the study design (systematic review and meta-analysis).

**Consent for publication:** Not applicable.

**Availability of data and materials:** All data generated or analyzed during this study are included in this article and its Supplemental Information Files.
