# Supplementary material for: Efficacy and safety of glucagon-like peptide-1 receptor agonists in Parkinson’s disease: a systematic review and meta-analysis of randomized placebo-controlled clinical trials
Source: Ther Adv Neurol Disord. 2026 Jan 31;19:17562864251408269. doi: 10.1177/17562864251408269 (PMC12861370; doi:10.1177/17562864251408269)
Supplement: sj-docx-1-tan-10.1177_17562864251408269 – Supplemental material for Efficacy and safety of glucagon-like peptide-1 receptor agonists in Parkinson’s disease: a systematic review and meta-analysis of randomized placebo-controlled clinical trials [file sj-docx-1-tan-10.1177_17562864251408269.docx]

**SUPPLEMENTAL MATERIAL**

**Complete search algorithm used in MEDLINE search.**

**Complete search algorithm used in SCOPUS search.**

**Supplementary Tables:**

Supplementary Table-S1. Table of excluded studies with reasons for exclusion.

**Supplementary Figures:**

Supplementary Figure-S1. Flowchart presenting the selection of eligible studies.

Supplementary Figure-S2. Traffic Light Plot presenting the quality assessment of included RCTs using the risk of bias in randomized trials (RoB 2) tool.

Supplementary Figure-S3. Quality assessment of included RCTs using the risk of bias in randomized trials (RoB2) tool, presented as percentages across all included studies.

Supplementary Figure-S4. Funnel Plot assessing Publication Bias among trials reporting changes in MDS-UPDRS Part III scores at “ON” state in patients with Parkinson’s disease treated with GLP-1 RAs versus placebo.

Supplementary Figure-S5. Funnel Plot assessing Publication Bias among trials reporting changes in MDS-UPDRS Part III scores at “OFF” state in patients with Parkinson’s disease treated with GLP-1 RAs versus placebo.

Supplementary Figure-S6. Forest plot comparing the changes in MDS-UPDRS Part I in patients with Parkinson’s disease treated with GLP-1 RAs versus placebo.

Supplementary Figure-S7. Funnel Plot assessing Publication Bias among trials reporting changes in MDS-UPDRS Part I in patients with Parkinson’s disease treated with GLP-1 RAs versus placebo.

Supplementary Figure-S8. Forest plot comparing the changes in MDS-UPDRS Part II in patients with Parkinson’s disease treated with GLP-1 RAs versus placebo.

Supplementary Figure-S9. Funnel Plot assessing Publication Bias among trials reporting changes in MDS-UPDRS Part II in patients with Parkinson’s disease treated with GLP-1 RAs versus placebo.

Supplementary Figure-S10. Forest plot comparing the changes in MDS-UPDRS Part IV in patients with Parkinson’s disease treated with GLP-1 RAs versus placebo.

Supplementary Figure-S11. Funnel Plot assessing Publication Bias among trials reporting changes in MDS-UPDRS Part IV in patients with Parkinson’s disease treated with GLP-1 RAs versus placebo.

Supplementary Figure-S12. Forest plot comparing the changes in the Non-Motor Symptoms Severity Scale at “ON” state in patients with Parkinson’s disease treated with GLP-1 RAs versus placebo.

Supplementary Figure-S13. Funnel Plot assessing Publication Bias among trials reporting changes in the Non-Motor Symptoms Severity Scale at “ON” state in patients with Parkinson’s disease treated with GLP-1 RAs versus placebo.

Supplementary Figure-S14. Forest plot comparing the changes in the MoCA score at “ON” state in patients with Parkinson’s disease treated with GLP-1 RAs versus placebo.

Supplementary Figure-S15. Funnel Plot assessing Publication Bias among trials reporting changes in the MoCA score at “ON” state in patients with Parkinson’s disease treated with GLP-1 RAs versus placebo.

Supplementary Figure-S16. Forest plot comparing the changes in the Parkinson’s Disease Questionnaire 39 at “ON” state in patients with Parkinson’s disease treated with GLP-1 RAs versus placebo.

Supplementary Figure-S17. Funnel Plot assessing Publication Bias among trials reporting changes in the Parkinson’s Disease Questionnaire 39 at “ON” state in patients with Parkinson’s disease treated with GLP-1 RAs versus placebo.

Supplementary Figure S-18. Forest plot comparing the risk of SAEs in patients with Parkinson’s disease treated with GLP-1 RAs versus placebo.

Supplementary Figure-S19. Forest plot comparing the risk of SAEs and AEs leading to treatment discontinuation in patients with Parkinson’s disease treated with GLP-1 RAs versus placebo.

Supplementary Figure-S20. Funnel Plot assessing Publication Bias among trials reporting the risk of SAEs and AEs leading to treatment discontinuation in patients with Parkinson’s disease treated with GLP-1 RAs versus placebo.

Supplementary Figure-S21. Funnel Plot assessing Publication Bias among trials reporting the risk of weight loss in patients with Parkinson’s disease treated with GLP-1 RAs versus placebo.

Supplementary Figure-S22. Forest plot comparing the risk of nausea in patients with Parkinson’s disease treated with GLP-1 RAs versus placebo.

Supplementary Figure-S23. Forest plot comparing the risk of vomiting in patients with Parkinson’s disease treated with GLP-1 RAs versus placebo.

Supplementary Figure-S24. Forest plot comparing the risk of diarrhoea in patients with Parkinson’s disease treated with GLP-1 RAs versus placebo.

Supplementary Figure-S25. Forest plot comparing the risk of constipation in patients with Parkinson’s disease treated with GLP-1 RAs versus placebo.

Supplementary Figure-S26. Forest plot comparing the risk of abdominal pain in patients with Parkinson’s disease treated with GLP-1 RAs versus placebo.

Supplementary Figure-S27. Sensitivity analysis using MD (95% CI): Forest plot comparing the changes in MDS-UPDRS Part III scores at “ON” state in patients with Parkinson’s disease treated with GLP-1 RAs versus placebo.

Supplementary Figure-S28. Sensitivity analysis using MD (95% CI): Forest plot comparing the changes in MDS-UPDRS Part III scores at “OFF” state in patients with Parkinson’s disease treated with GLP-1 RAs versus placebo.

Supplementary Figure-S29. Sensitivity analysis using MD (95% CI): Forest plot comparing the changes in MDS-UPDRS Part I in patients with Parkinson’s disease treated with GLP-1 RAs versus placebo.

Supplementary Figure-S30. Sensitivity analysis using MD (95% CI): Forest plot comparing the changes in MDS-UPDRS Part II in patients with Parkinson’s disease treated with GLP-1 RAs versus placebo.

Supplementary Figure-S31. Sensitivity analysis using MD (95% CI): Forest plot comparing the changes in MDS-UPDRS Part IV in patients with Parkinson’s disease treated with GLP-1 RAs versus placebo.

Supplementary Figure-S32. Sensitivity analysis using MD (95% CI): Forest plot comparing the changes in the Non-Motor Symptoms Severity Scale at “ON” state in patients with Parkinson’s disease treated with GLP-1 RAs versus placebo.

Supplementary Figure-S33. Sensitivity analysis using MD (95% CI): Forest plot comparing the changes in the MoCA score at “ON” state in patients with Parkinson’s disease treated with GLP-1 RAs versus placebo.

Supplementary Figure-S34. Sensitivity analysis using MD (95% CI): Forest plot comparing the changes in the Parkinson’s Disease Questionnaire 39 at “ON” state in patients with Parkinson’s disease treated with GLP-1 RAs versus placebo.

**ANALYSIS**

**Expanded Methods**

**Complete search algorithm used in MEDLINE search**

((((((((glucagon-like peptide-1 receptor agonist) OR (lixisenatide)) OR (exenatide)) OR (liraglutide)) OR (semaglutide)) OR (albiglutide)) OR (dulaglutide)) OR (NLY01)) AND ((Parkinson)) AND ((randomized controlled trial) OR (randomised controlled trial))

**Complete search algorithm used in SCOPUS search**

((((((((glucagon-like peptide-1 receptor agonist) OR (lixisenatide)) OR (exenatide)) OR (liraglutide)) OR (semaglutide)) OR (albiglutide)) OR (dulaglutide)) OR (NLY01)) AND ((Parkinson)) AND ((randomized controlled trial) OR (randomised controlled trial))

**Supplementary Tables**

**Table-S1.** Table of excluded studies with reasons for exclusion.

| **Reason for exclusion** | **PMID (PubMed identifier)** |
| --- | --- |
| Commentary | 28777758 |
| No outcomes of interest | 30640362 |
| Non-placebo-controlled RCT | 24662192 |
| Non-placebo-controlled RCT | 23728174 |

**Supplementary Figures**

**Figure S1.** Flowchart presenting the selection of eligible studies.

**
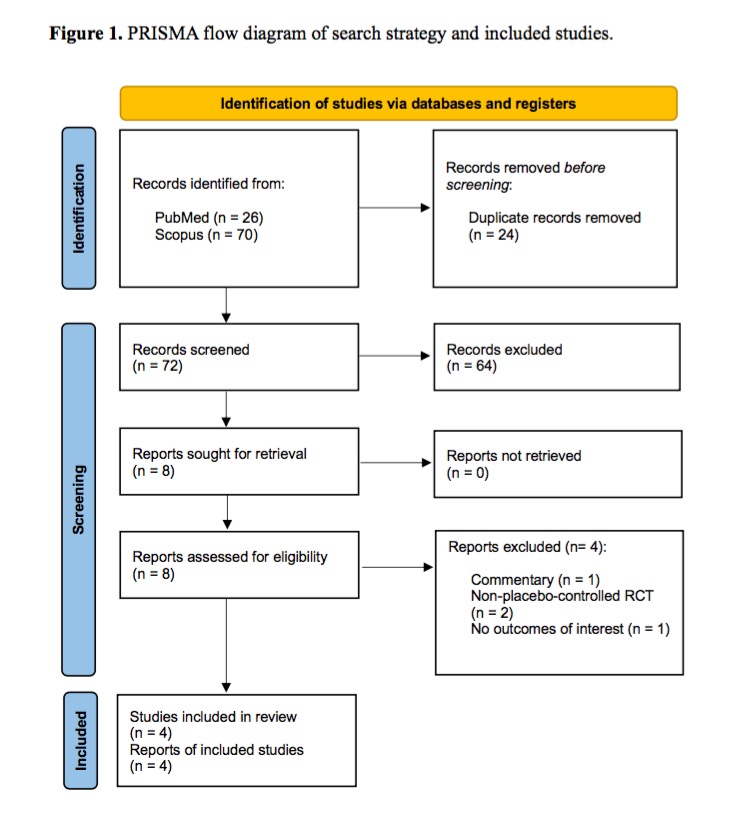
**

**Figure S2.** Traffic Light Plot presenting the quality assessment of included RCTs^1-4^ using the risk of bias in randomized trials (RoB 2) tool.^5^

**Figure S3.** Quality assessment of included RCTs using the risk of bias in randomized trials (RoB2) tool, presented as percentages across all included studies.


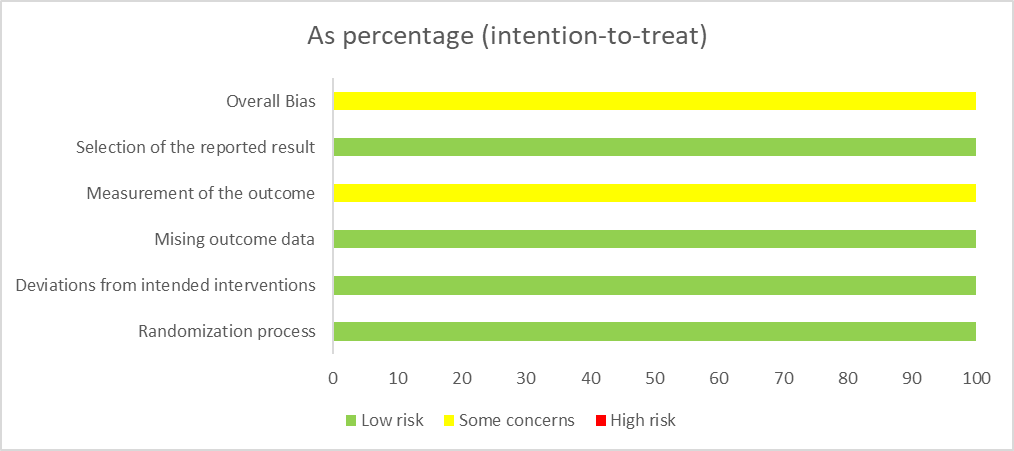


**Figure S4.** Funnel Plot assessing Publication Bias among trials reporting changes in MDS-UPDRS Part III scores at “ON” state in patients with Parkinson’s disease treated with GLP-1 RAs versus placebo.


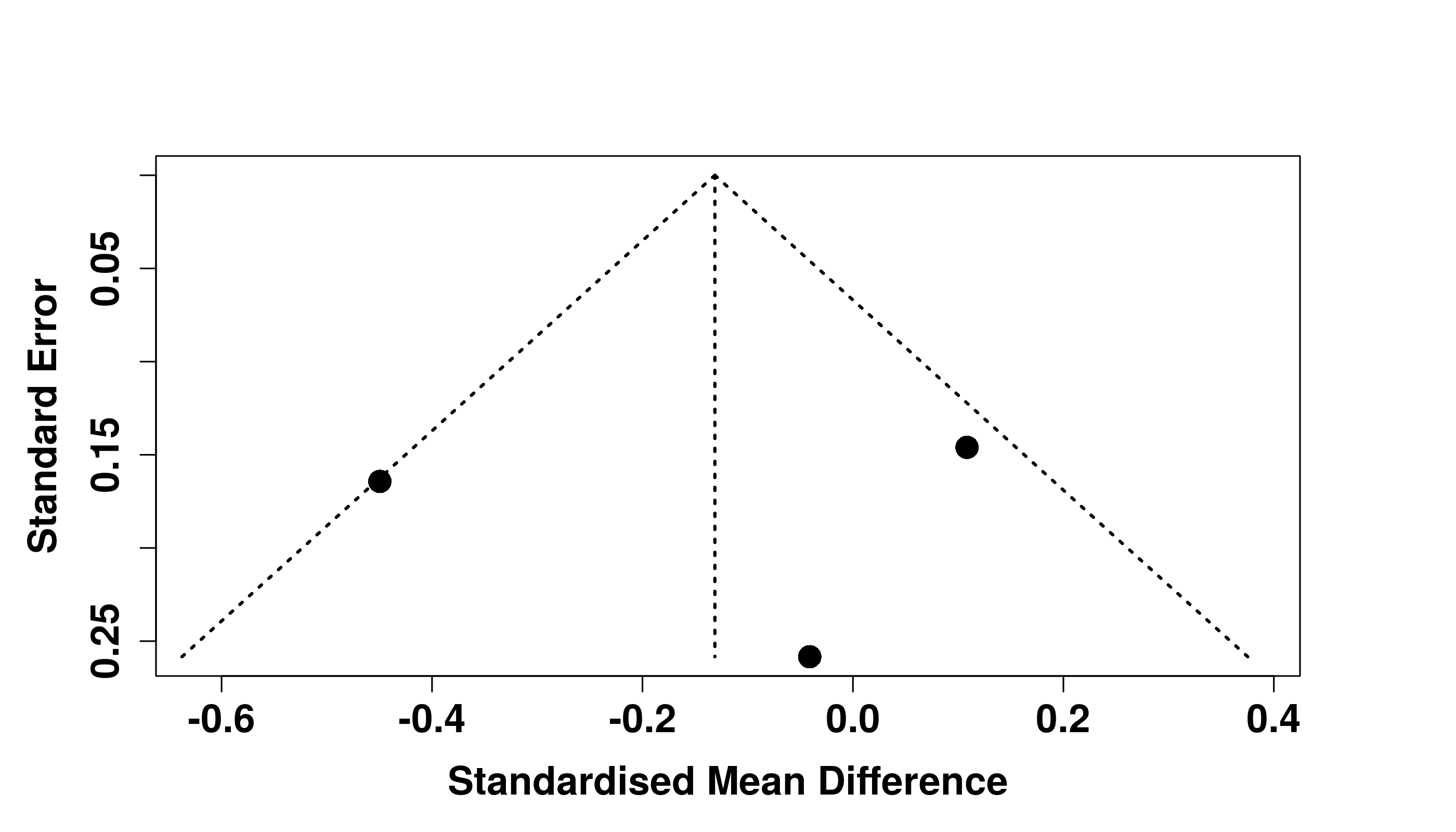


**Figure S5.** Funnel Plot assessing Publication Bias among trials reporting changes in MDS-

UPDRS Part III scores at “OFF” state in patients with Parkinson’s disease treated with GLP-1 RAs versus placebo.


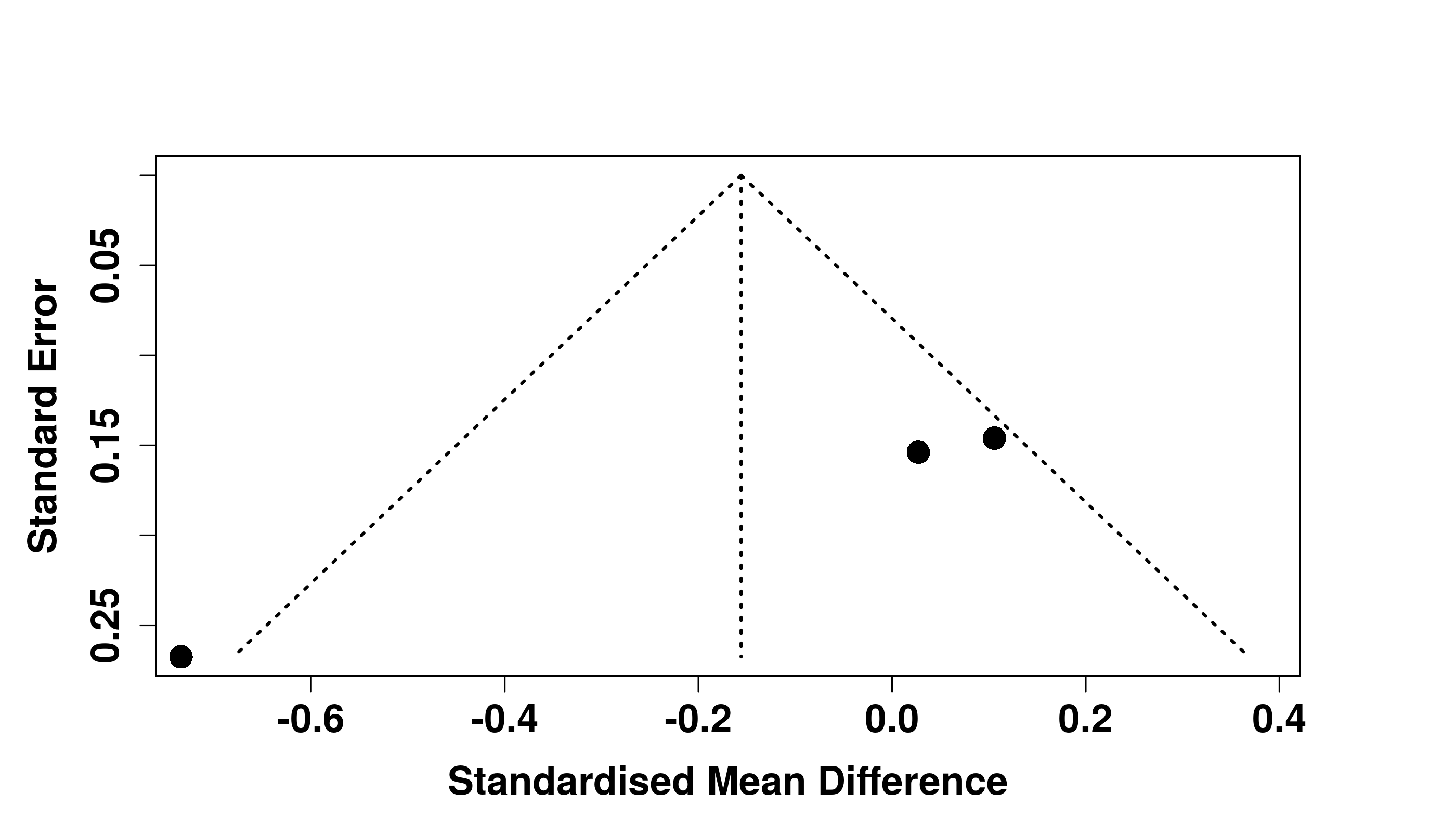


**Figure S6.** Forest plot comparing the changes in MDS-UPDRS Part I in patients with Parkinson’s disease treated with GLP-1 RAs versus placebo.


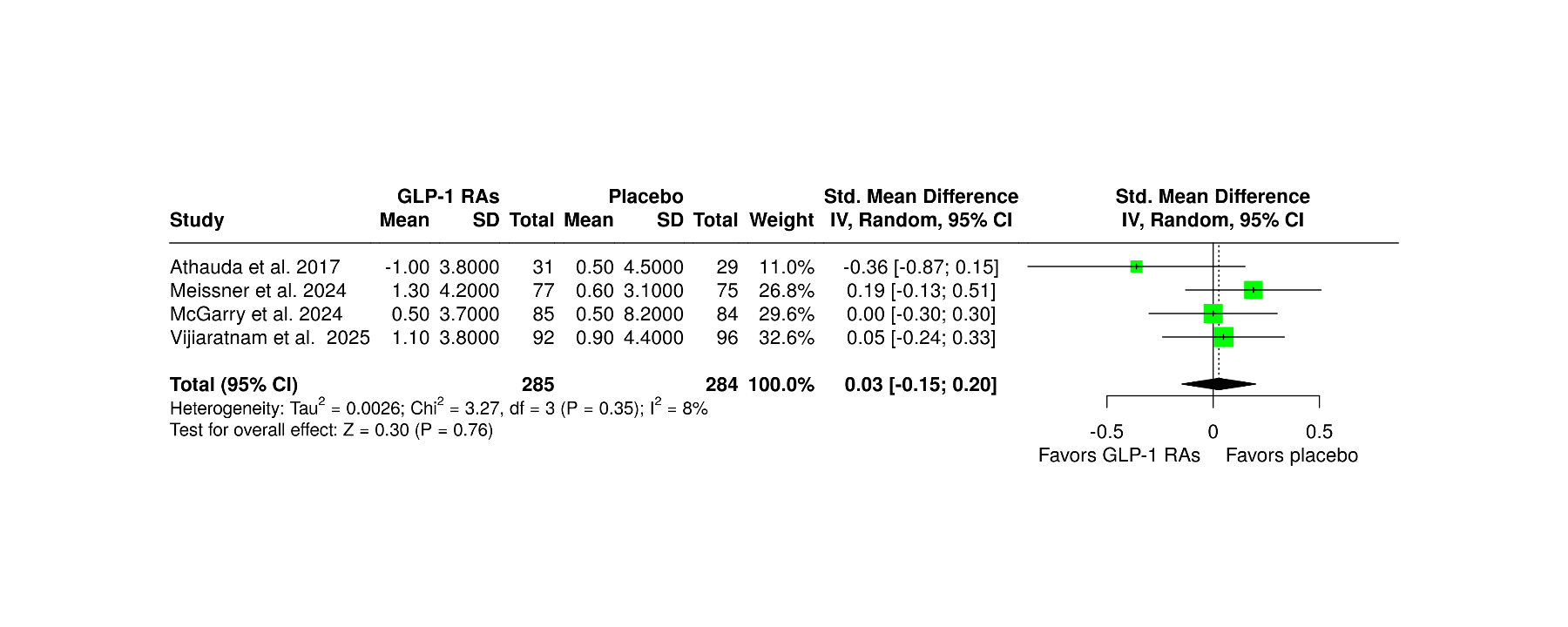


**Figure S7.** Funnel Plot assessing Publication Bias among trials reporting changes in MDS-UPDRS Part I in patients with Parkinson’s disease treated with GLP-1 RAs versus placebo.


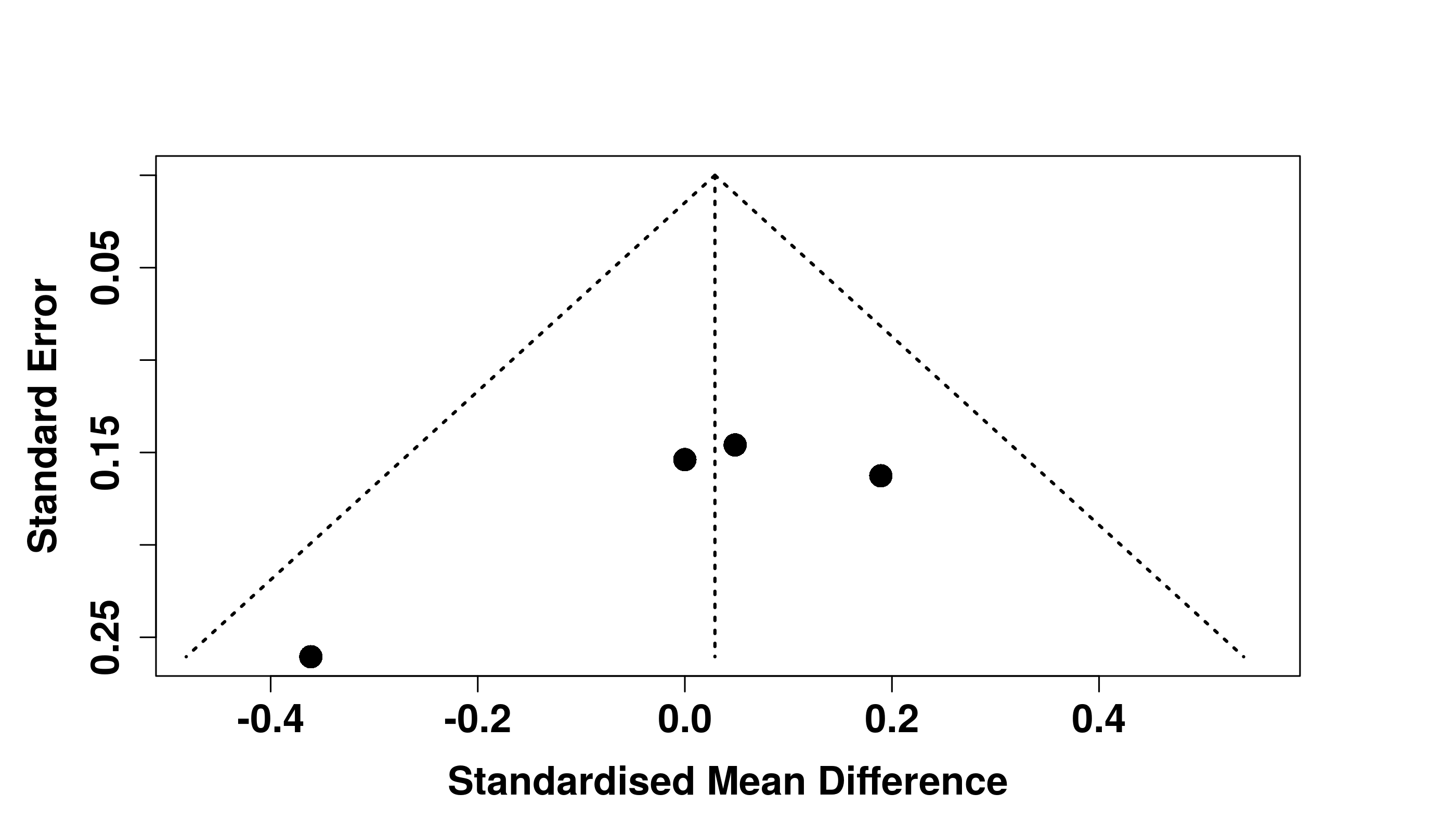


**Figure S8.** Forest plot comparing the changes in MDS-UPDRS Part II in patients with Parkinson’s disease treated with GLP-1 RAs versus placebo.


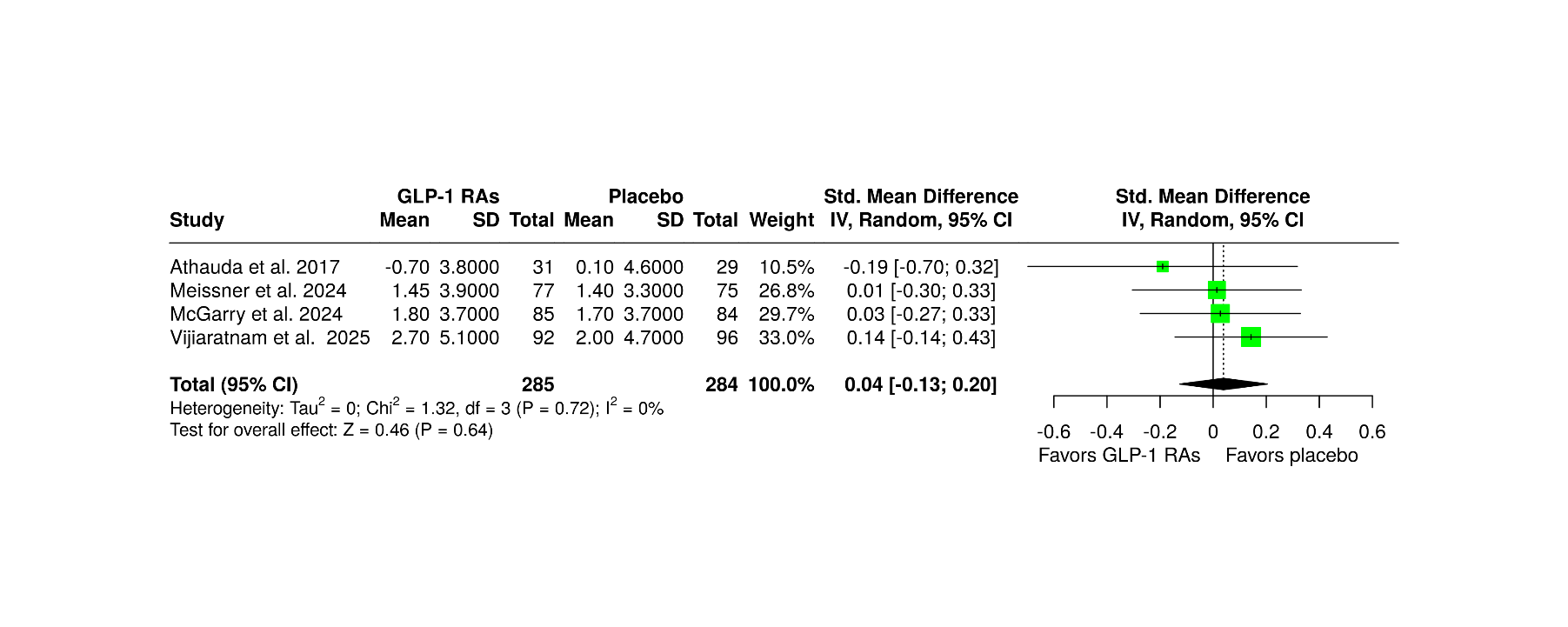


**Figure S9.** Funnel Plot assessing Publication Bias among trials reporting changes in MDS-UPDRS Part II in patients with Parkinson’s disease treated with GLP-1 RAs versus placebo.


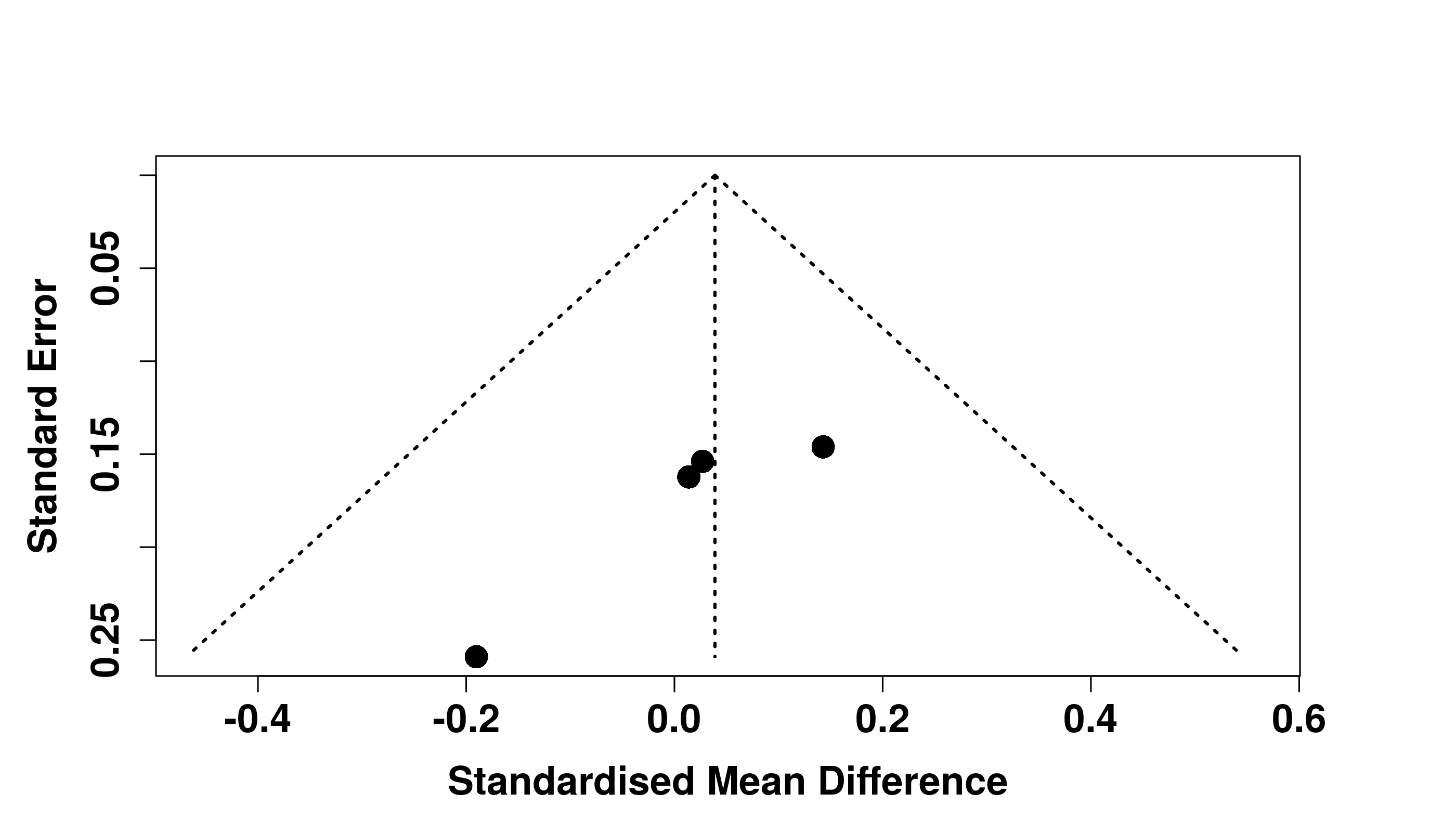


**Figure S10.** Forest plot comparing the changes in MDS-UPDRS Part IV in patients with Parkinson’s disease treated with GLP-1 RAs versus placebo.


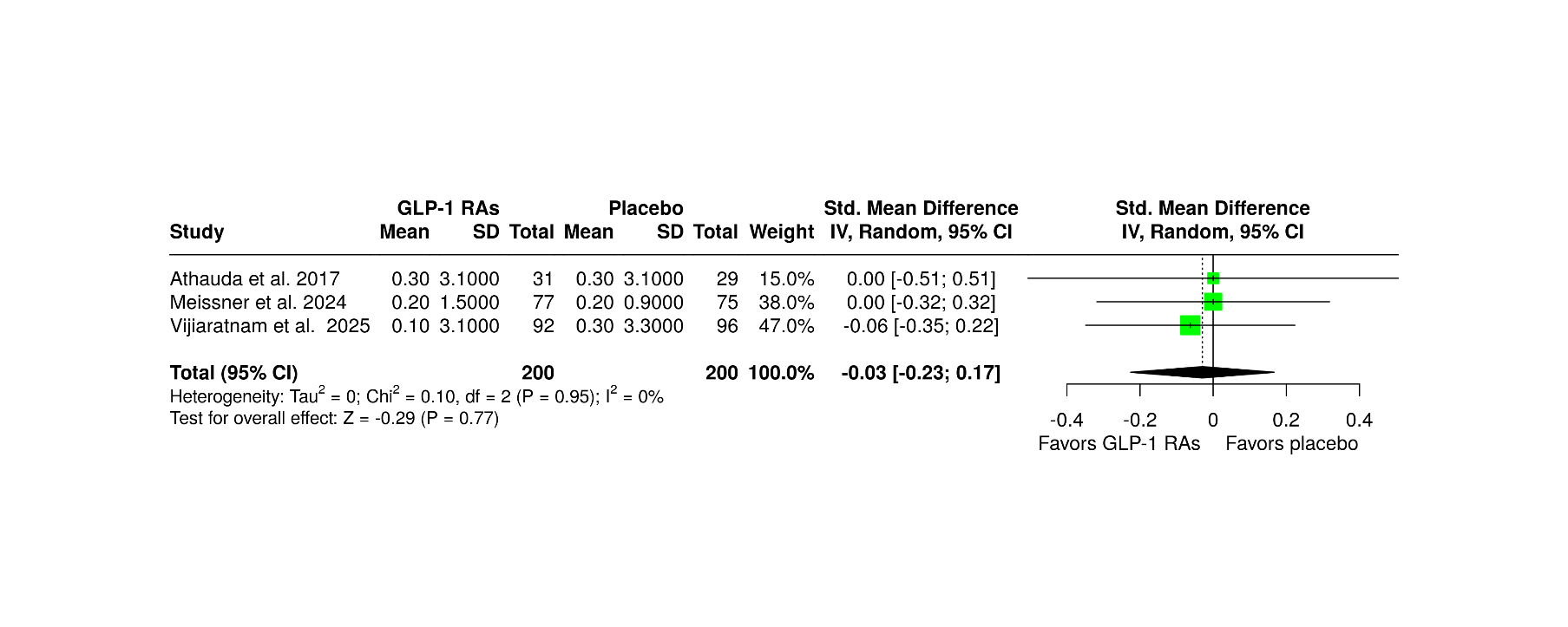


**Figure S11.** Funnel Plot assessing Publication Bias among trials reporting changes in MDS-UPDRS Part IV in patients with Parkinson’s disease treated with GLP-1 RAs versus placebo.


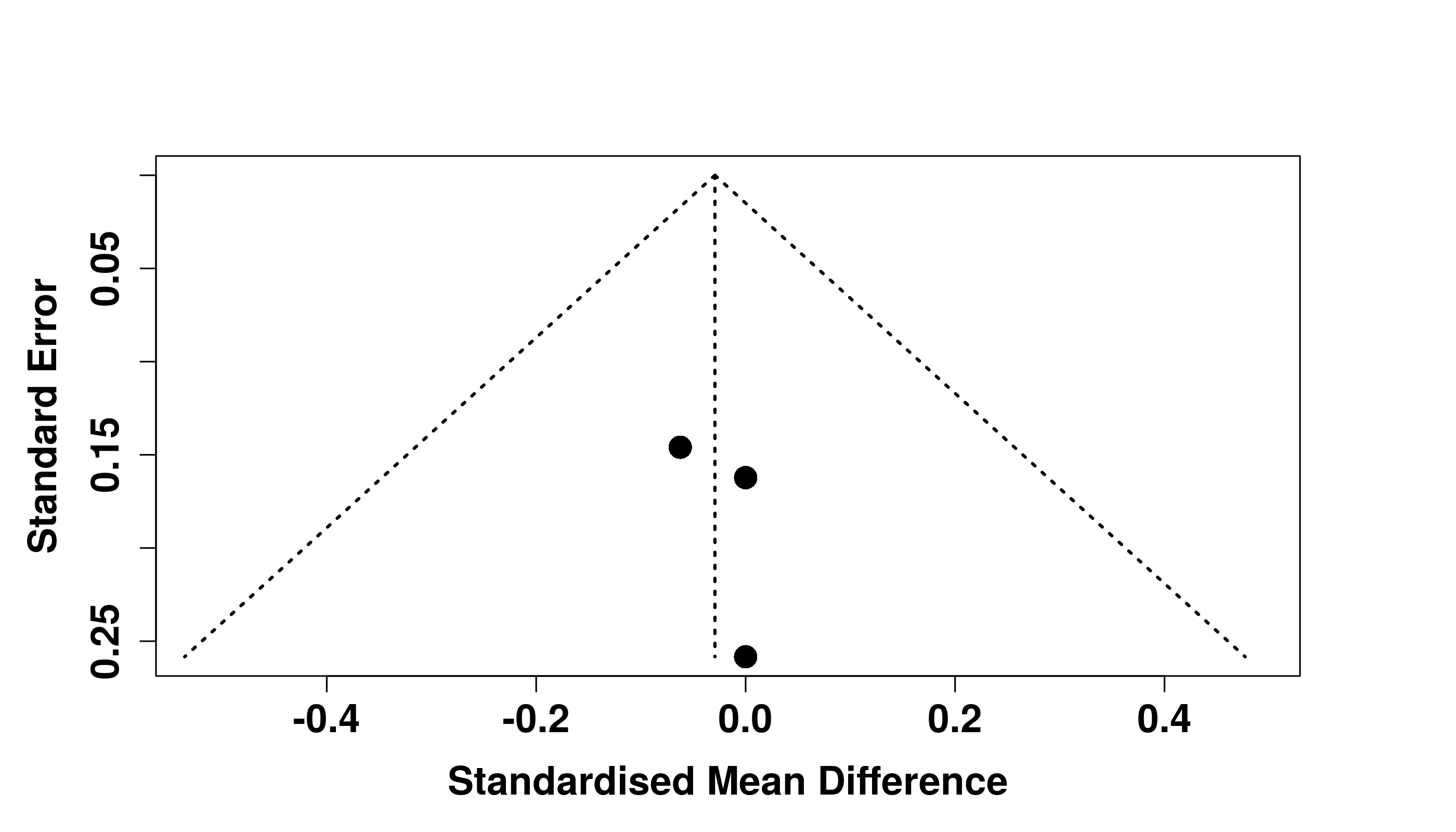


**Figure S12.** Forest plot comparing the changes in the Non-Motor Symptoms Severity Scale at “ON” state in patients with Parkinson’s disease treated with GLP-1 RAs versus placebo.


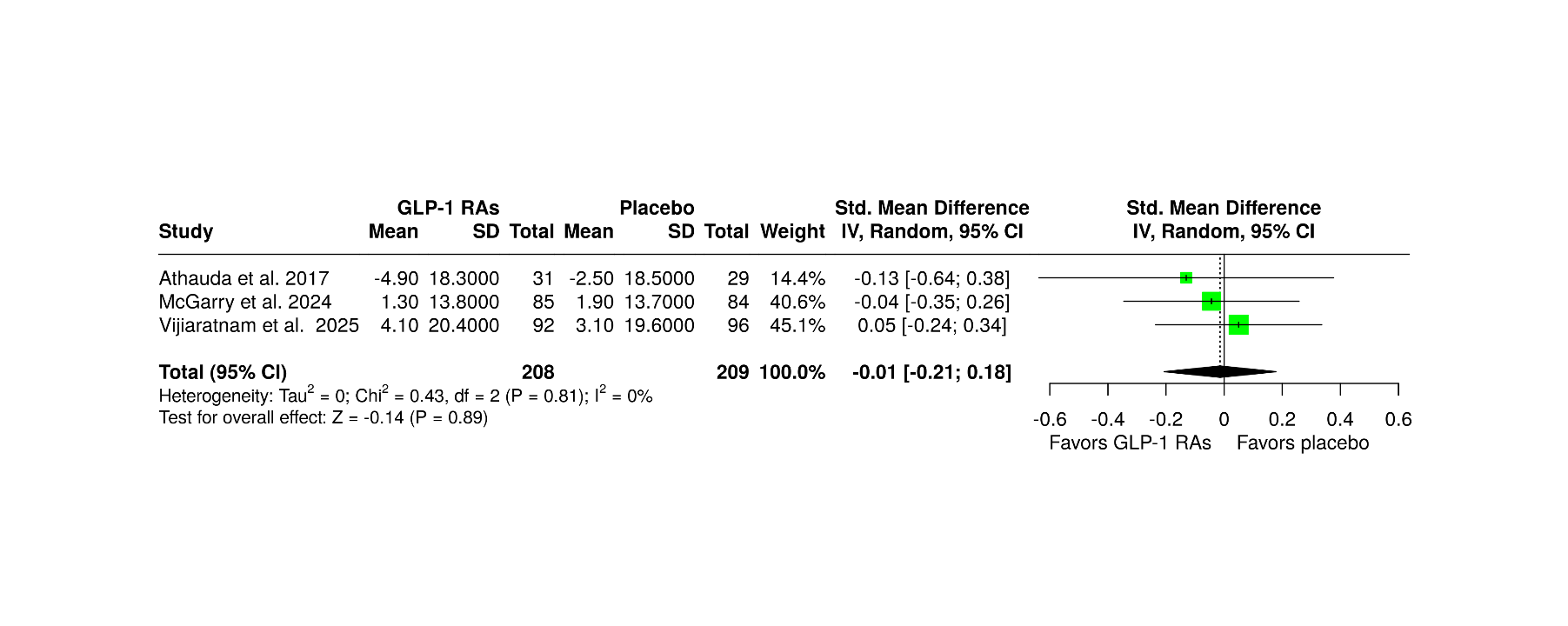


**Figure S13.** Funnel Plot assessing Publication Bias among trials reporting changes in the Non-Motor Symptoms Severity Scale at “ON” state in patients with Parkinson’s disease treated with GLP-1 RAs versus placebo.


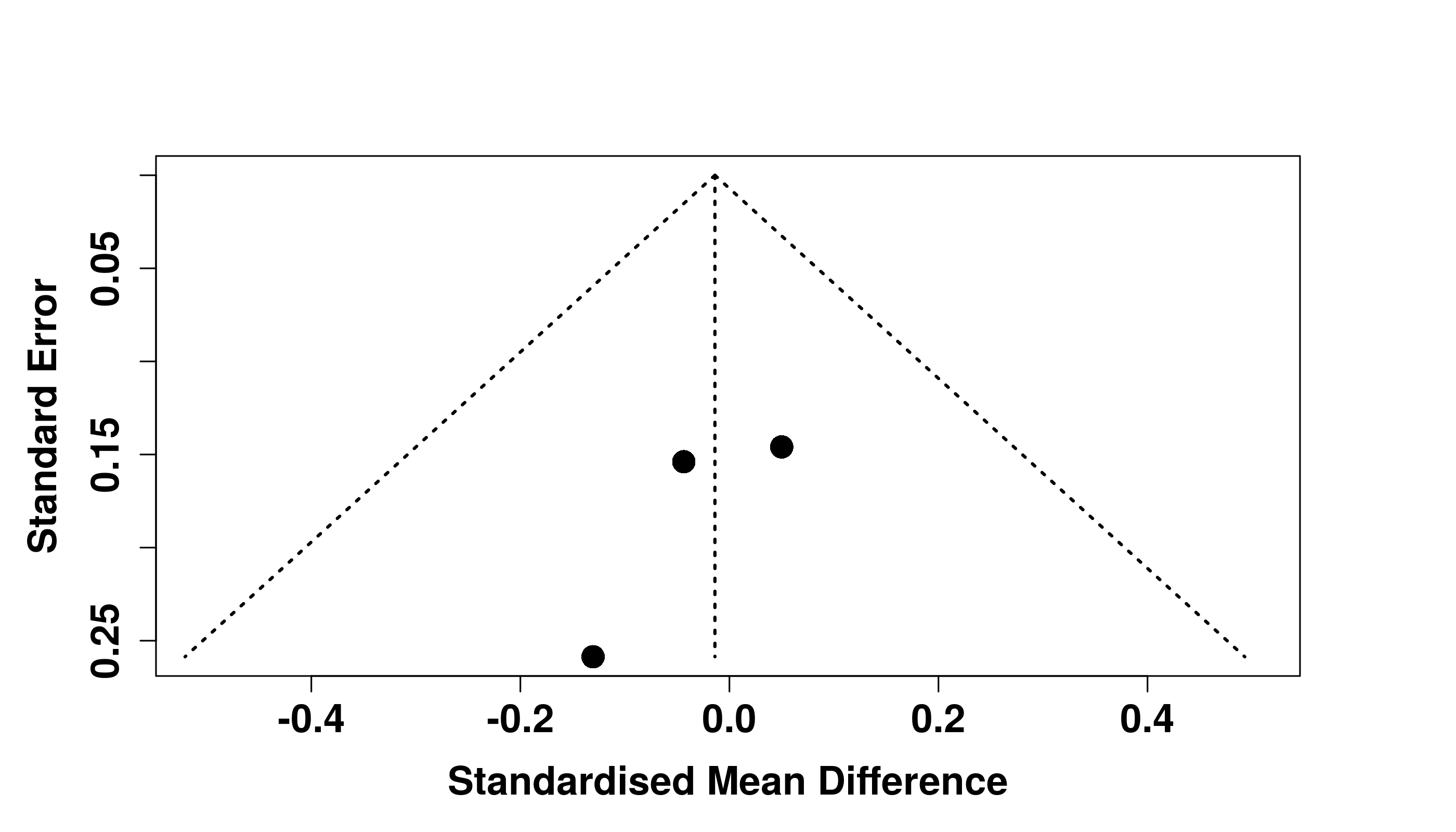


**Figure S14.** Forest plot comparing the changes in the MoCA score at “ON” state in patients with Parkinson’s disease treated with GLP-1 RAs versus placebo.


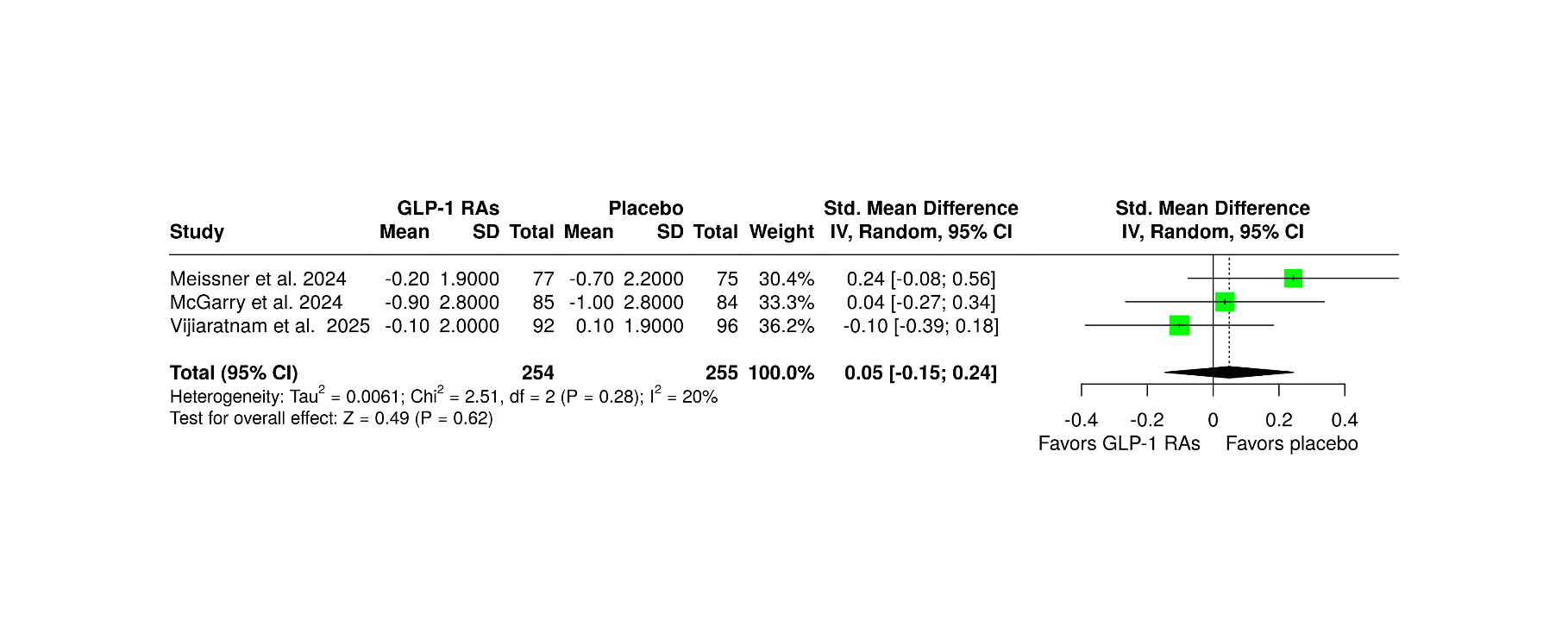


**Figure S15.** Funnel Plot assessing Publication Bias among trials reporting changes in the MoCA score at “ON” state in patients with Parkinson’s disease treated with GLP-1 RAs versus placebo.


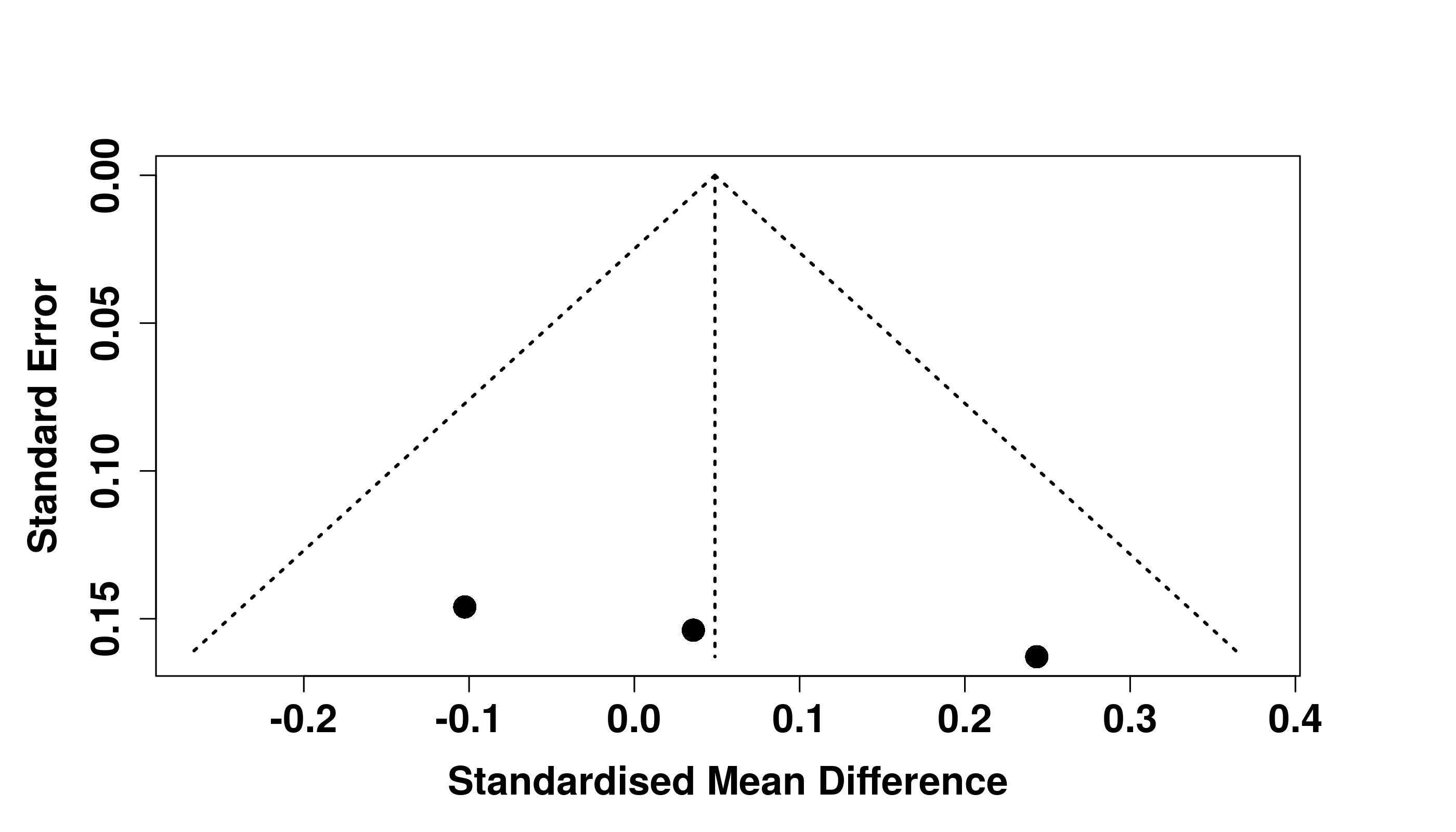


**Figure S16.** Forest plot comparing the changes in the Parkinson’s Disease Questionnaire 39 at “ON” state in patients with Parkinson’s disease treated with GLP-1 RAs versus placebo.


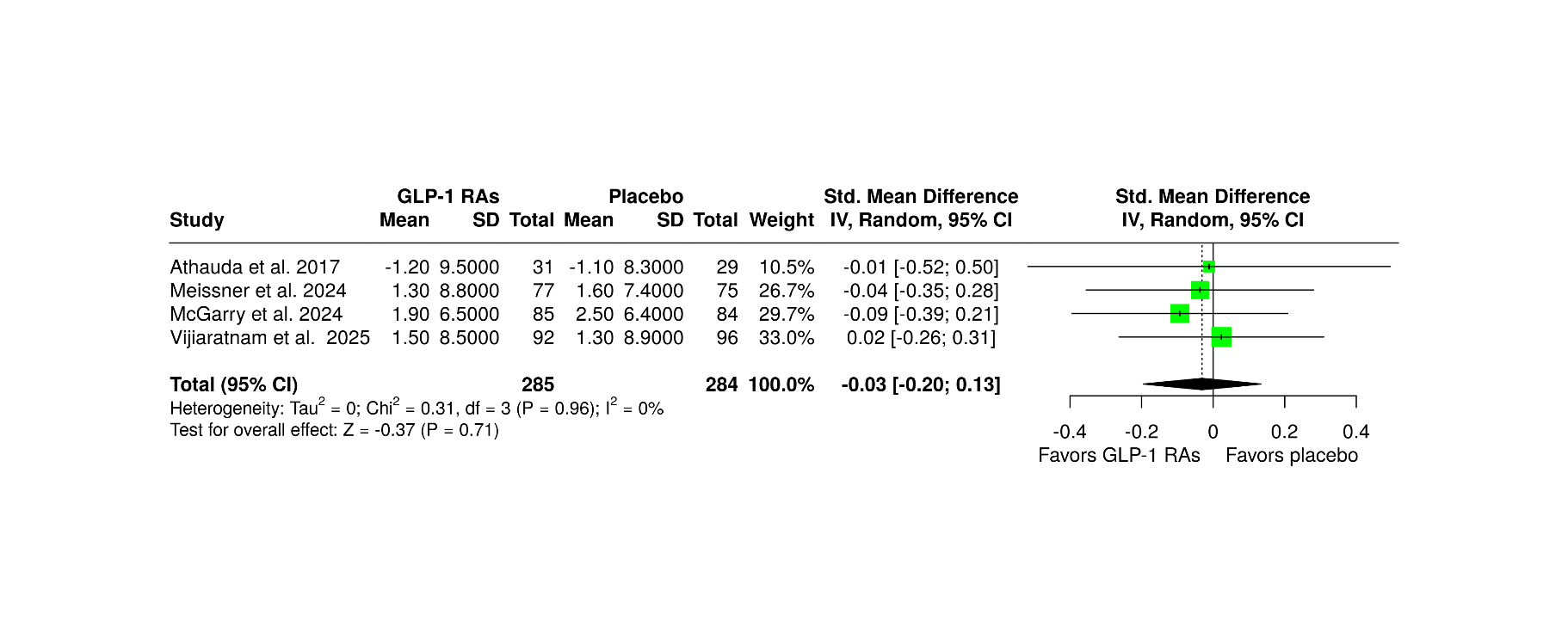


**Figure S17.** Funnel Plot assessing Publication Bias among trials reporting changes in the Parkinson’s Disease Questionnaire 39 at “ON” state in patients with Parkinson’s disease treated with GLP-1 RAs versus placebo.


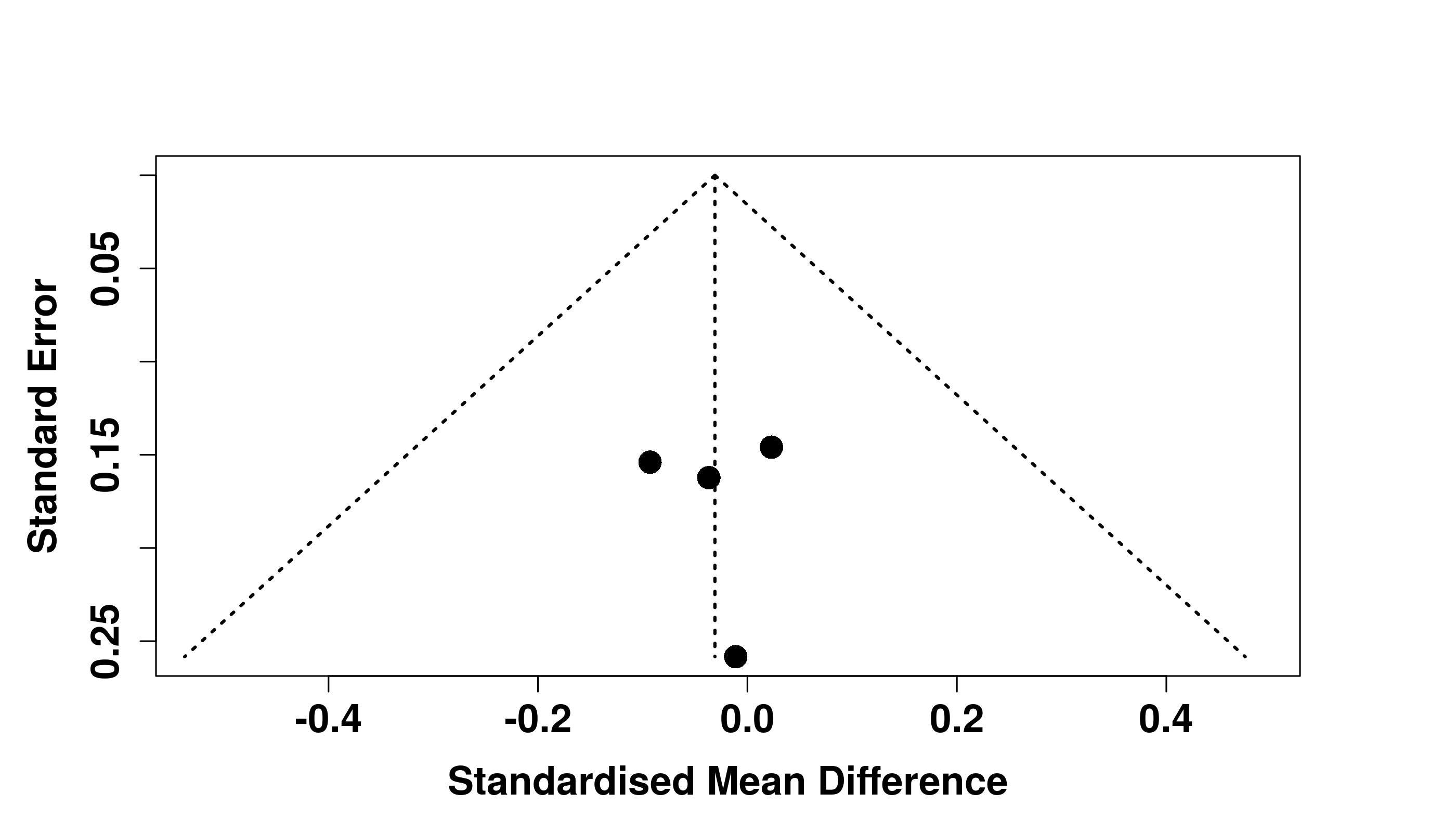


**Figure S18.** Forest plot comparing the risk of SAEs in patients with Parkinson’s disease treated with GLP-1 RAs versus placebo.

**
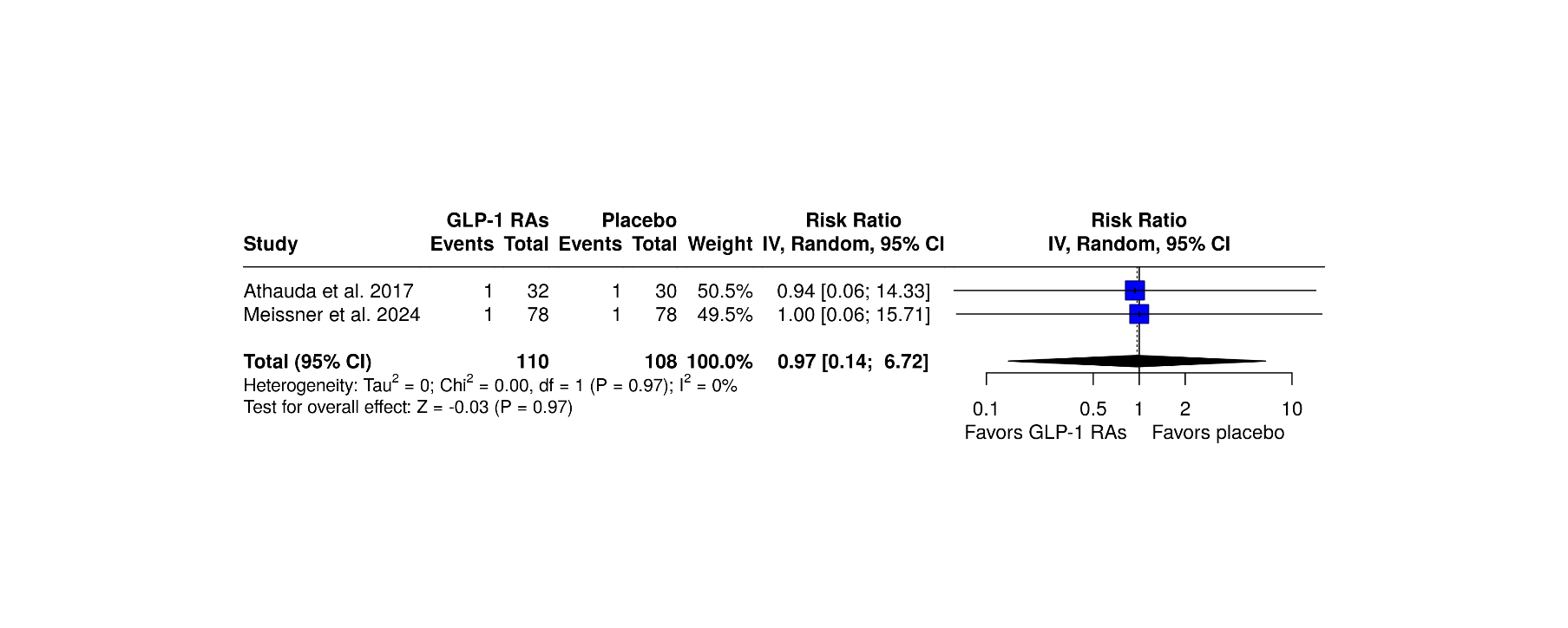
**

**Figure S19.** Forest plot comparing the risk of SAEs and AEs leading to treatment discontinuation in patients with Parkinson’s disease treated with GLP-1 RAs versus placebo.


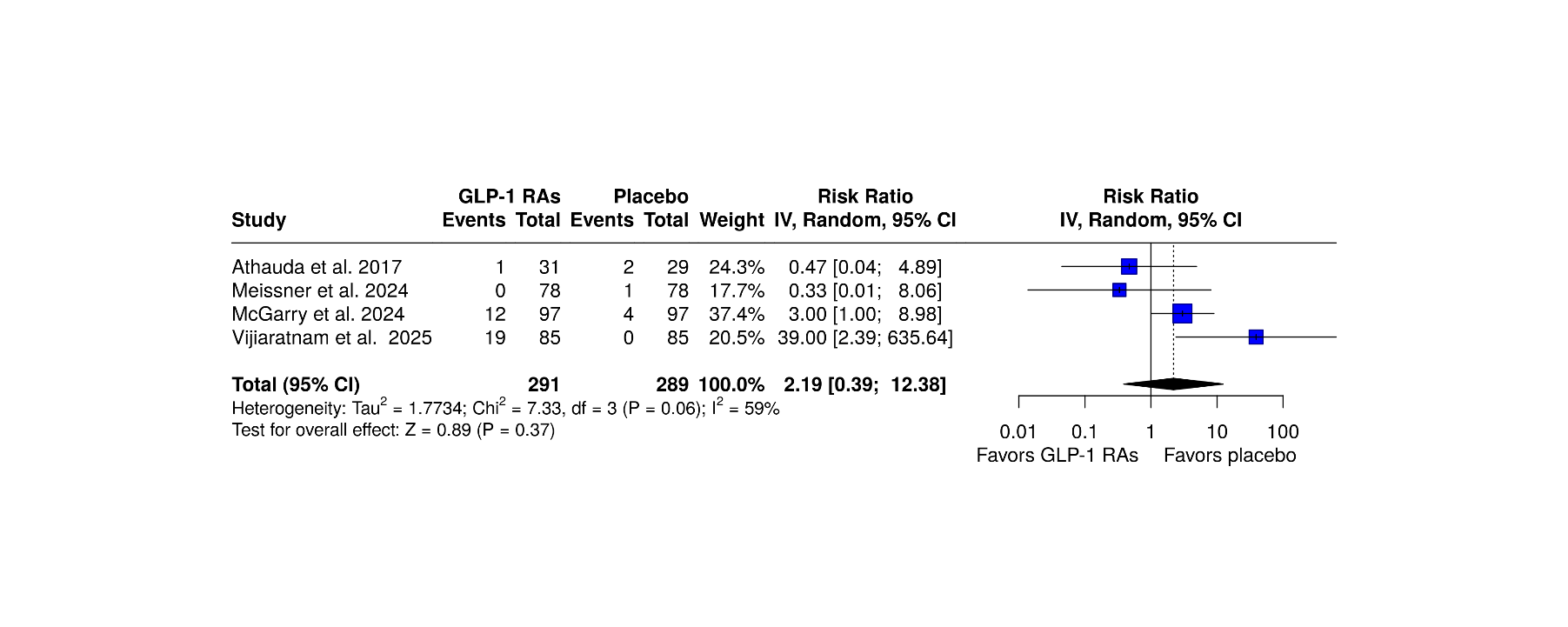


**Figure S20.** Funnel Plot assessing Publication Bias among trials reporting the risk of SAEs and AEs leading to treatment discontinuation in patients with Parkinson’s disease treated with GLP-1 RAs versus placebo.


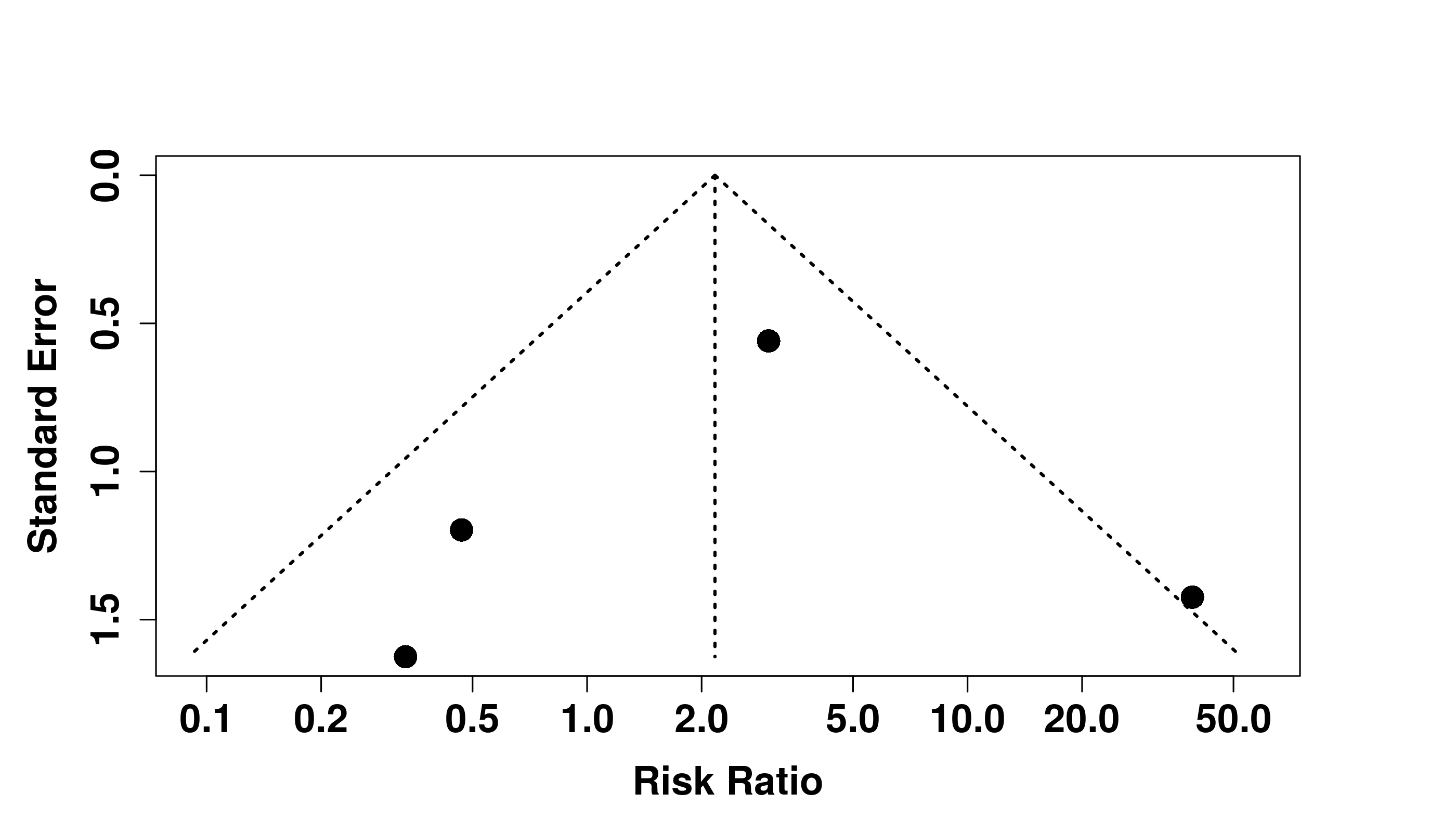


**Figure S21.** Funnel Plot assessing Publication Bias among trials reporting the risk of weight loss in patients with Parkinson’s disease treated with GLP-1 RAs versus placebo.


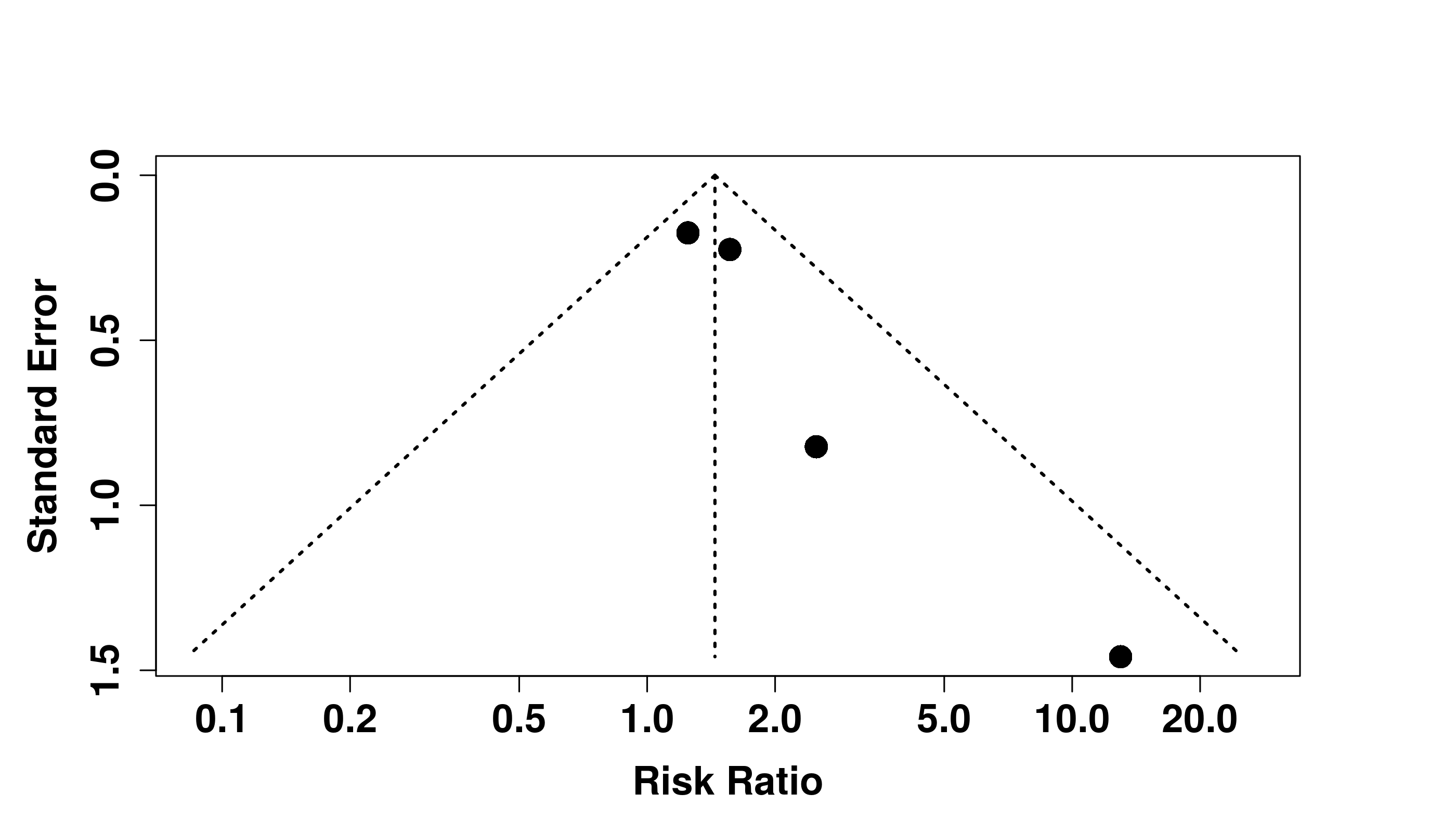


**Figure S22.** Forest plot comparing the risk of nausea in patients with Parkinson’s disease treated with GLP-1 RAs versus placebo.


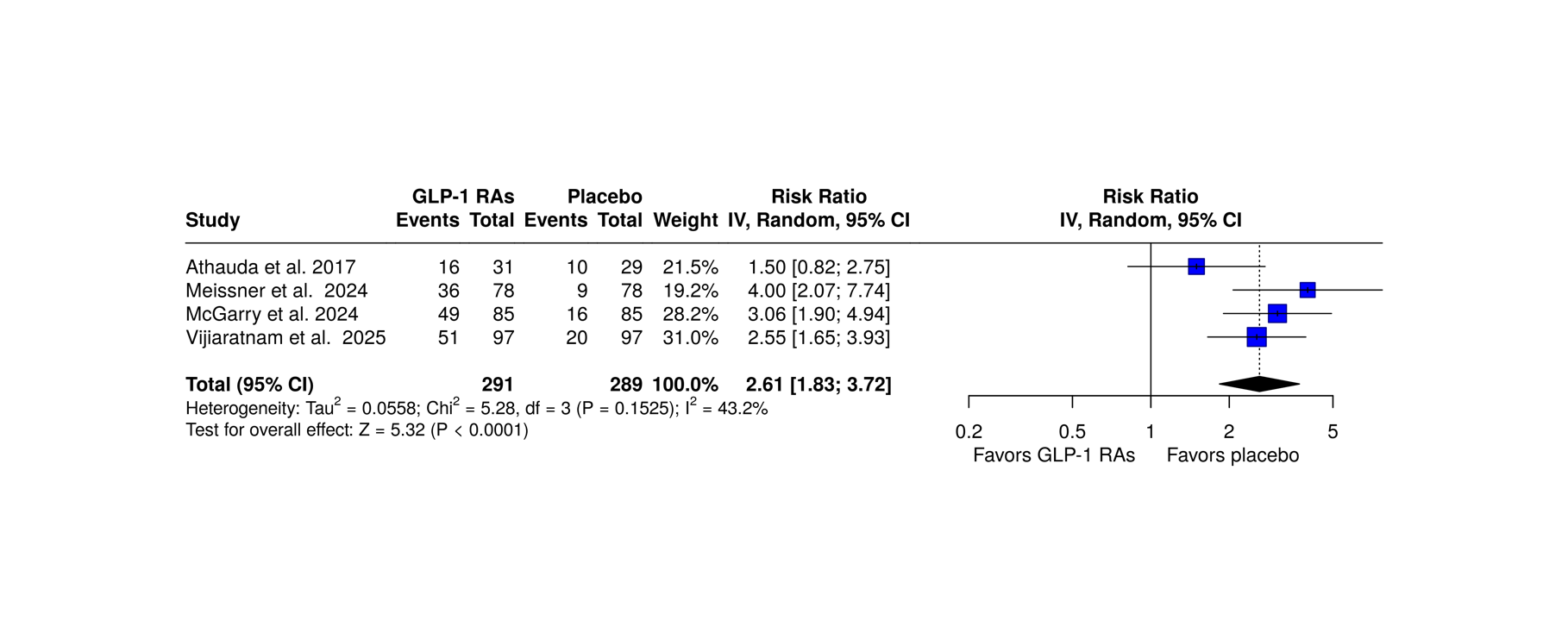


**Figure S23.** Forest plot comparing the risk of vomiting in patients with Parkinson’s disease treated with GLP-1 RAs versus placebo.

**
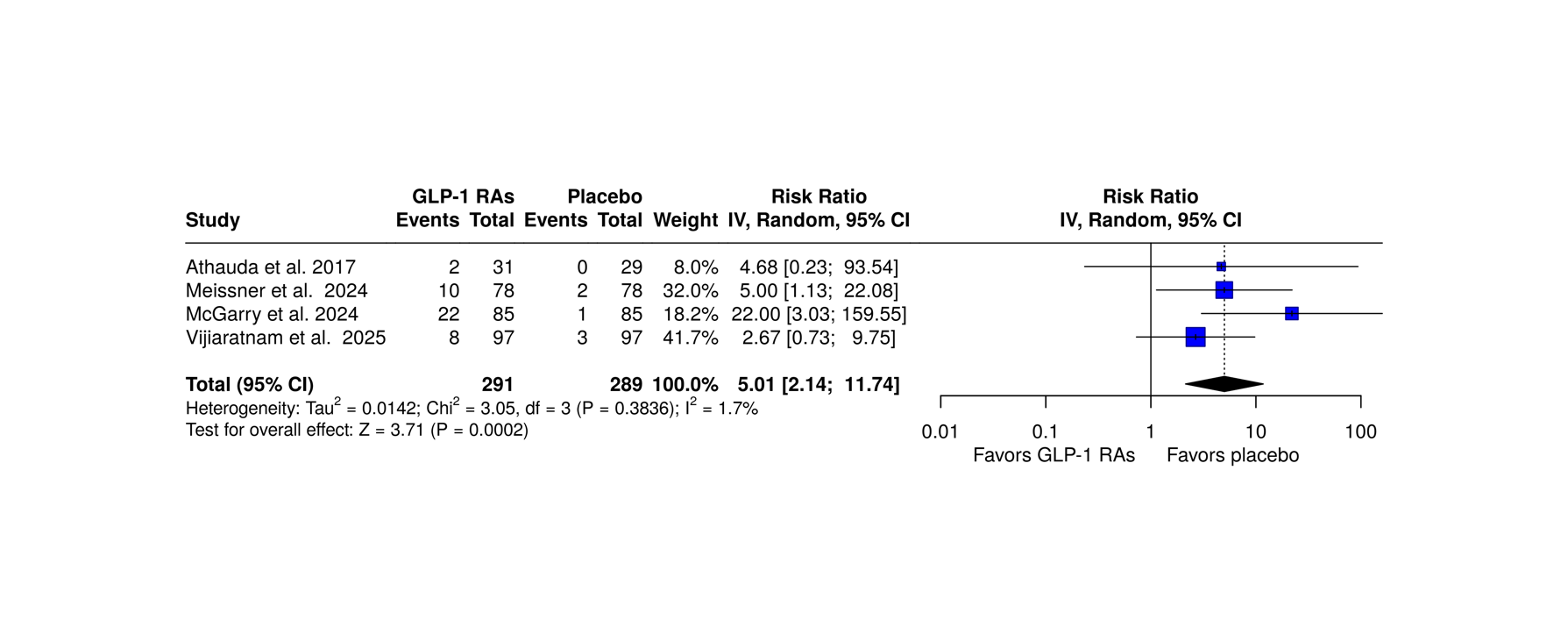
**

**Figure S24.** Forest plot comparing the risk of diarrhoea in patients with Parkinson’s disease treated with GLP-1 RAs versus placebo.

**
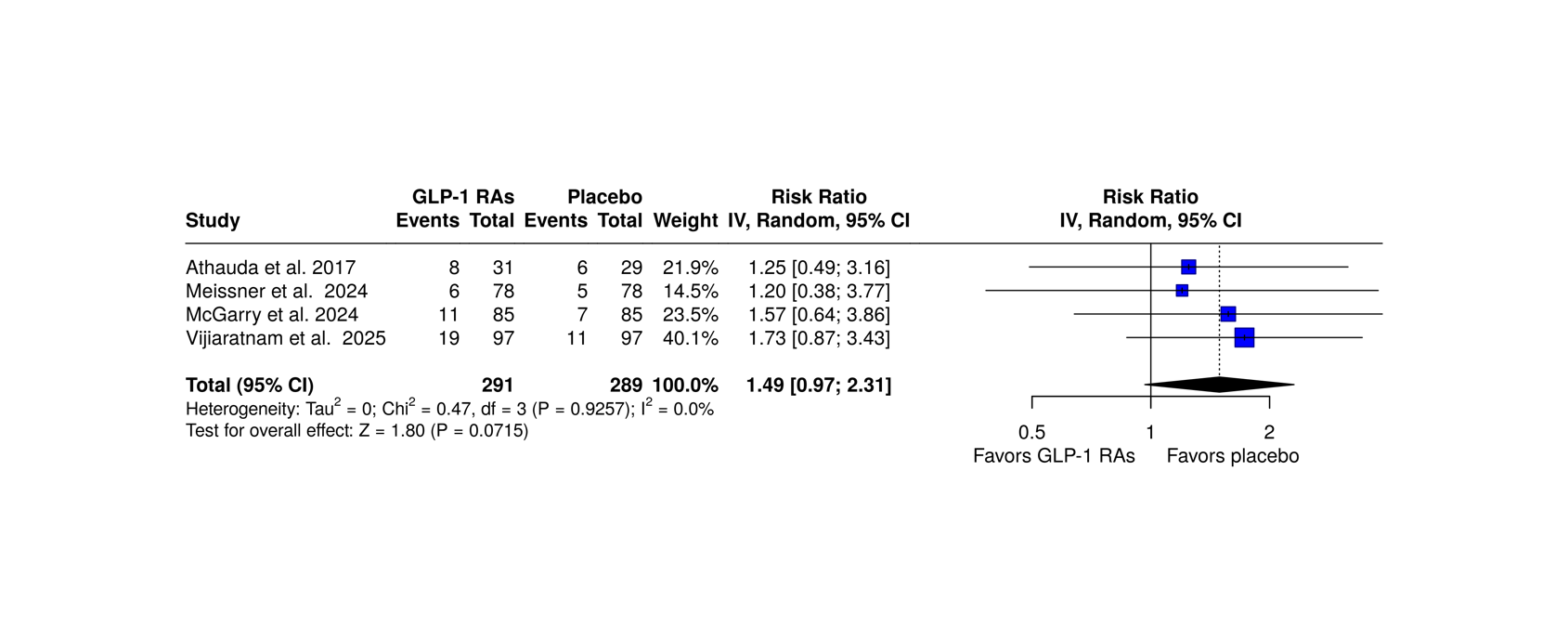
**

**Figure S25.** Forest plot comparing the risk of constipation in patients with Parkinson’s disease treated with GLP-1 RAs versus placebo.

**
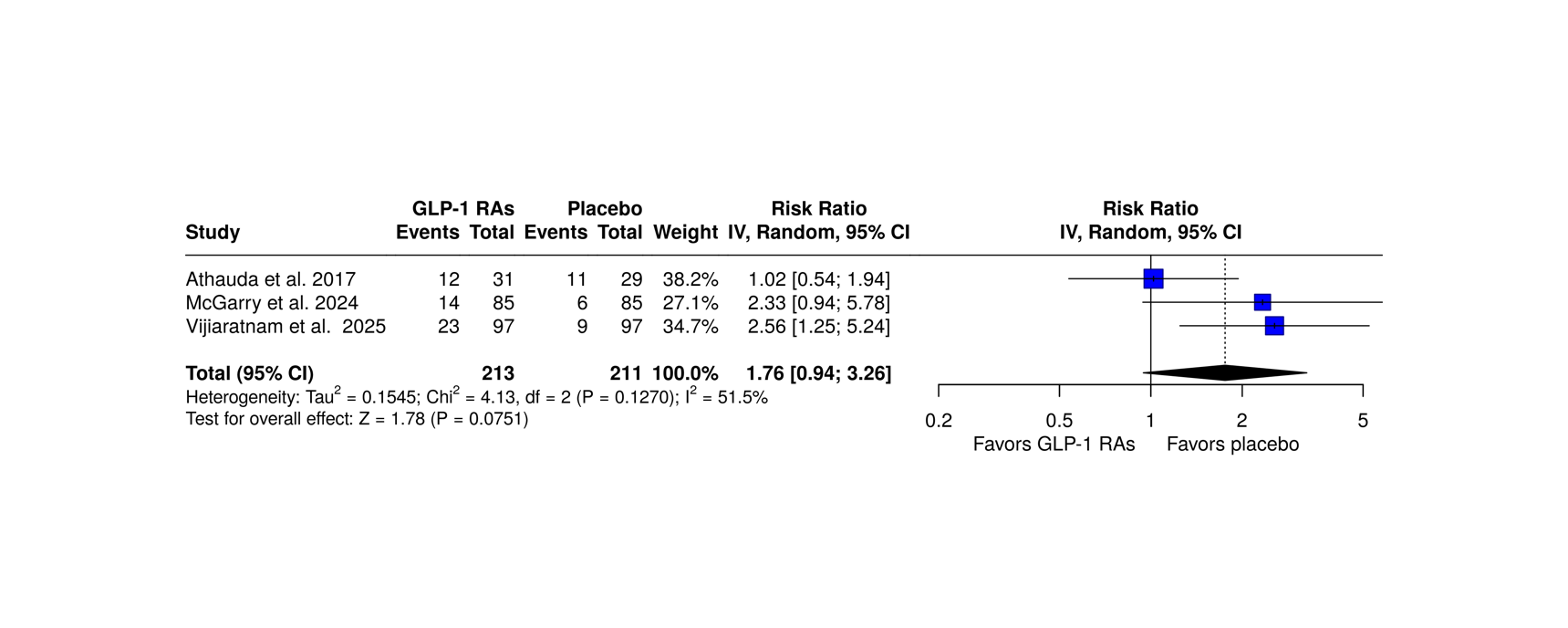
**

**Figure S26.** Forest plot comparing the risk of abdominal pain in patients with Parkinson’s disease treated with GLP-1 RAs versus placebo.

**
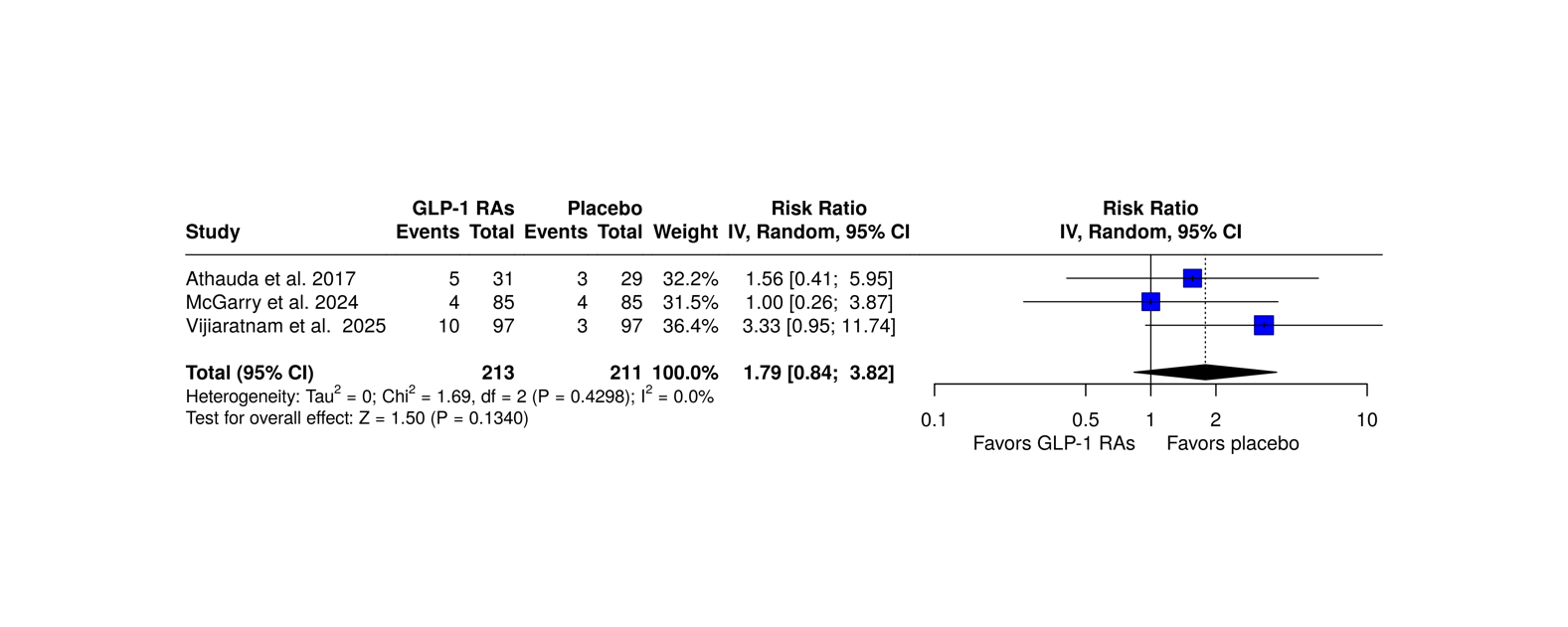
**


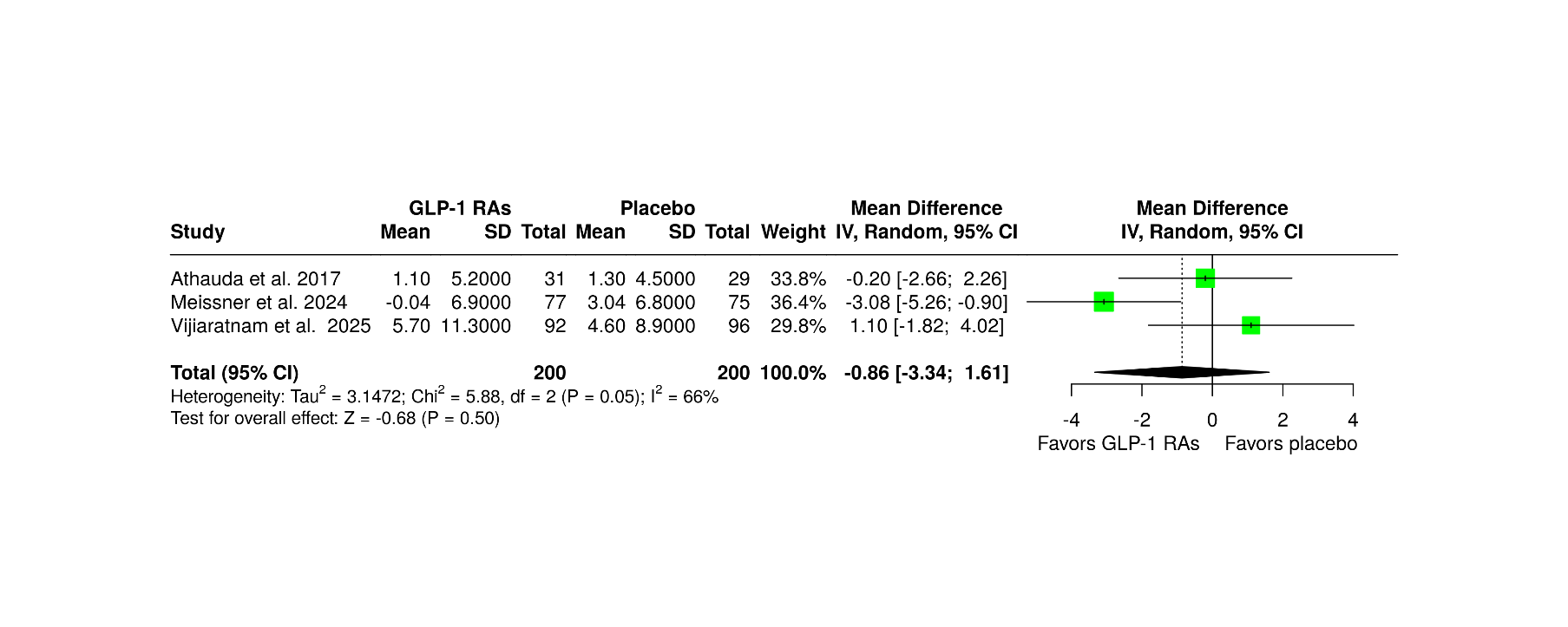
**Figure S27.** Sensitivity analysis using MD (95% CI): Forest plot comparing the changes in MDS-UPDRS Part III scores at “ON” state in patients with Parkinson’s disease treated with GLP-1 RAs versus placebo.


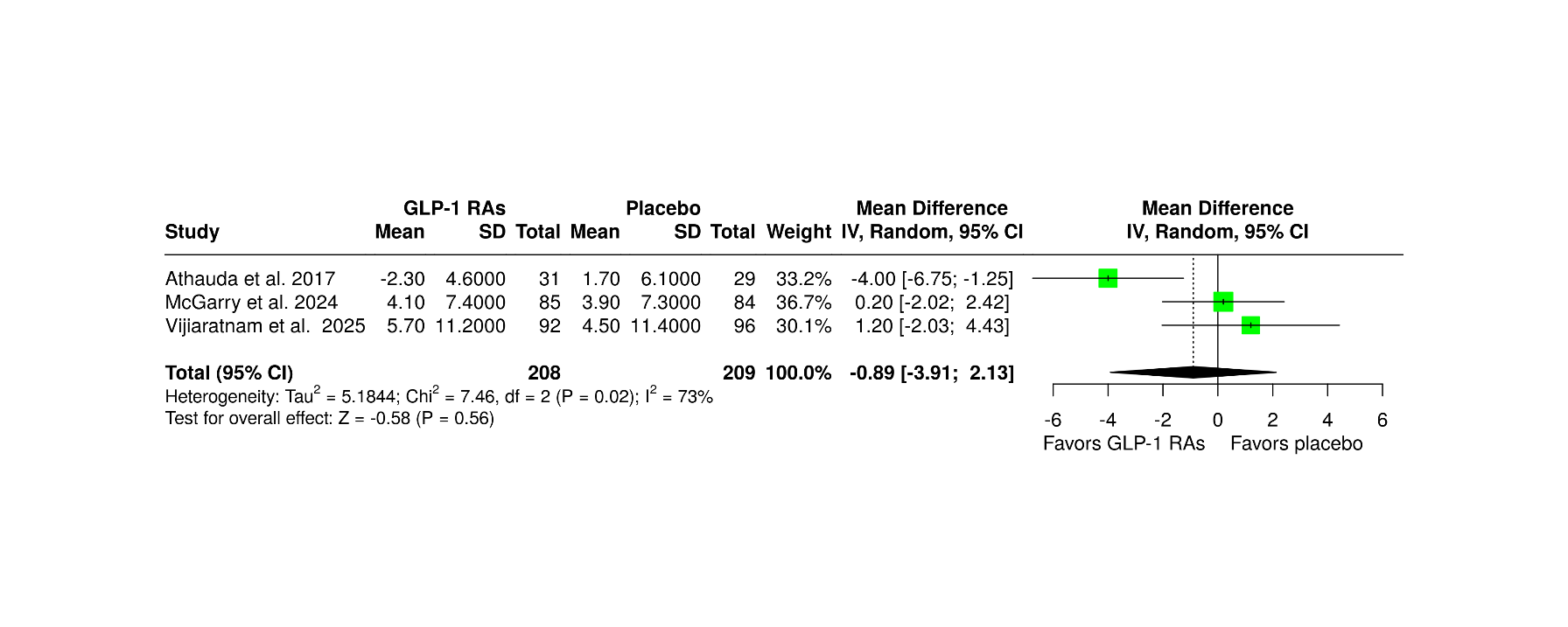
**Figure S28.** Sensitivity analysis using MD (95% CI): Forest plot comparing the changes in MDS-UPDRS Part III scores at “OFF” state in patients with Parkinson’s disease treated with GLP-1 RAs versus placebo.


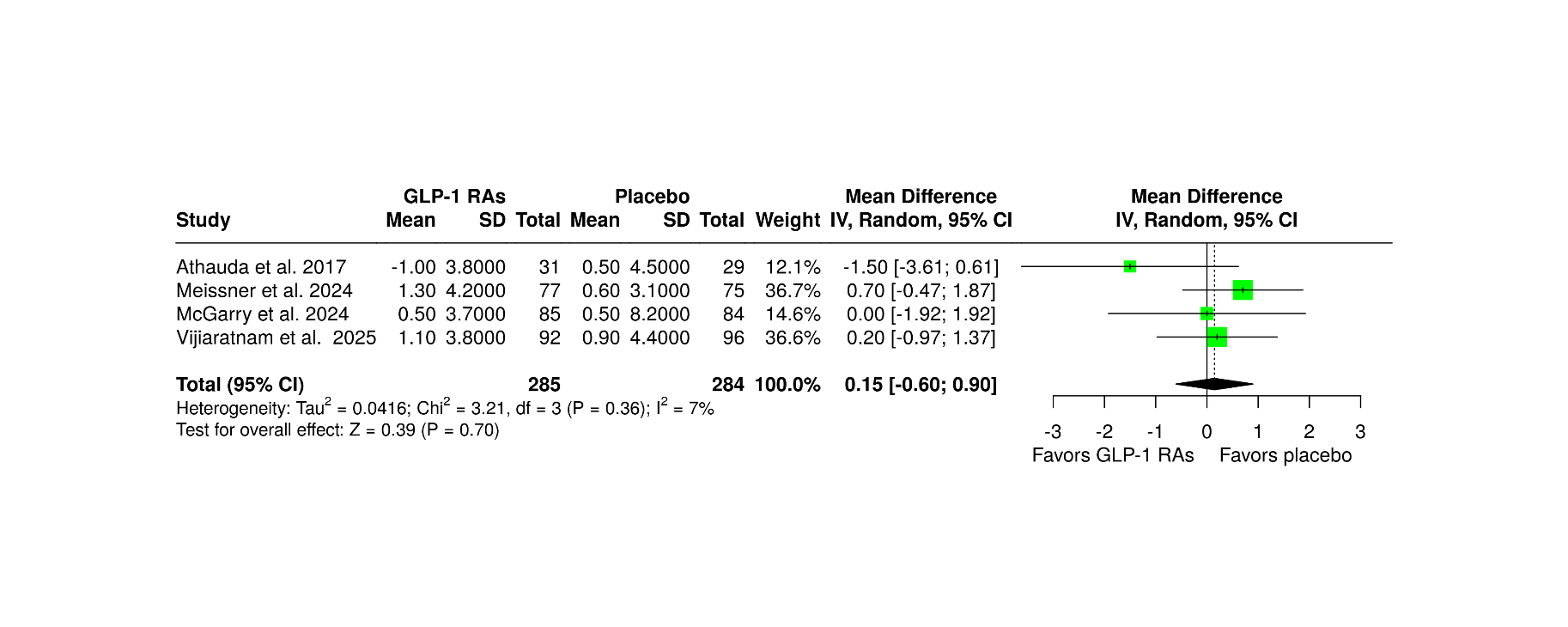
**Figure S29.** Sensitivity analysis using MD (95% CI): Forest plot comparing the changes in MDS-UPDRS Part I in patients with Parkinson’s disease treated with GLP-1 RAs versus placebo.


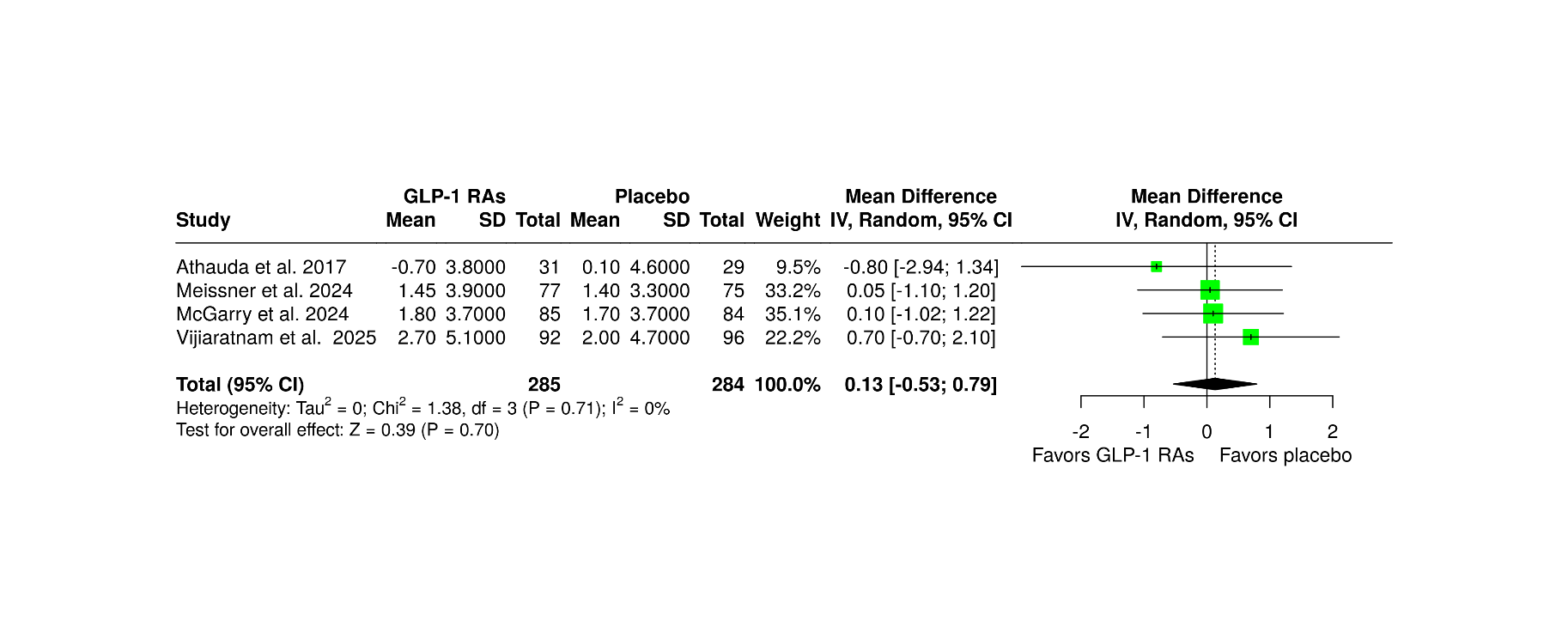
**Figure S30.** Sensitivity analysis using MD (95% CI): Forest plot comparing the changes in MDS-UPDRS Part II in patients with Parkinson’s disease treated with GLP-1 RAs versus placebo.

**Figure S31.** Sensitivity analysis using MD (95% CI): Forest plot comparing the changes in MDS-UPDRS Part IV in patients with Parkinson’s disease treated with GLP-1 RAs versus placebo.


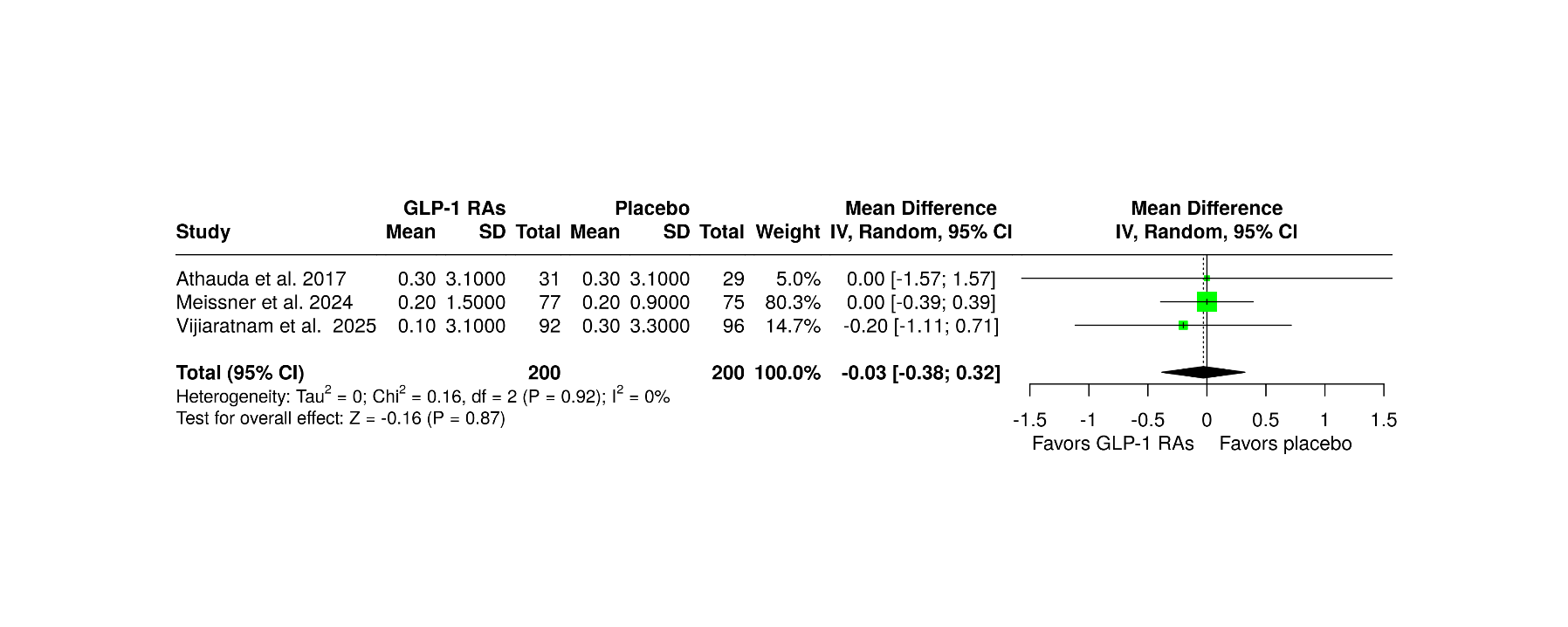


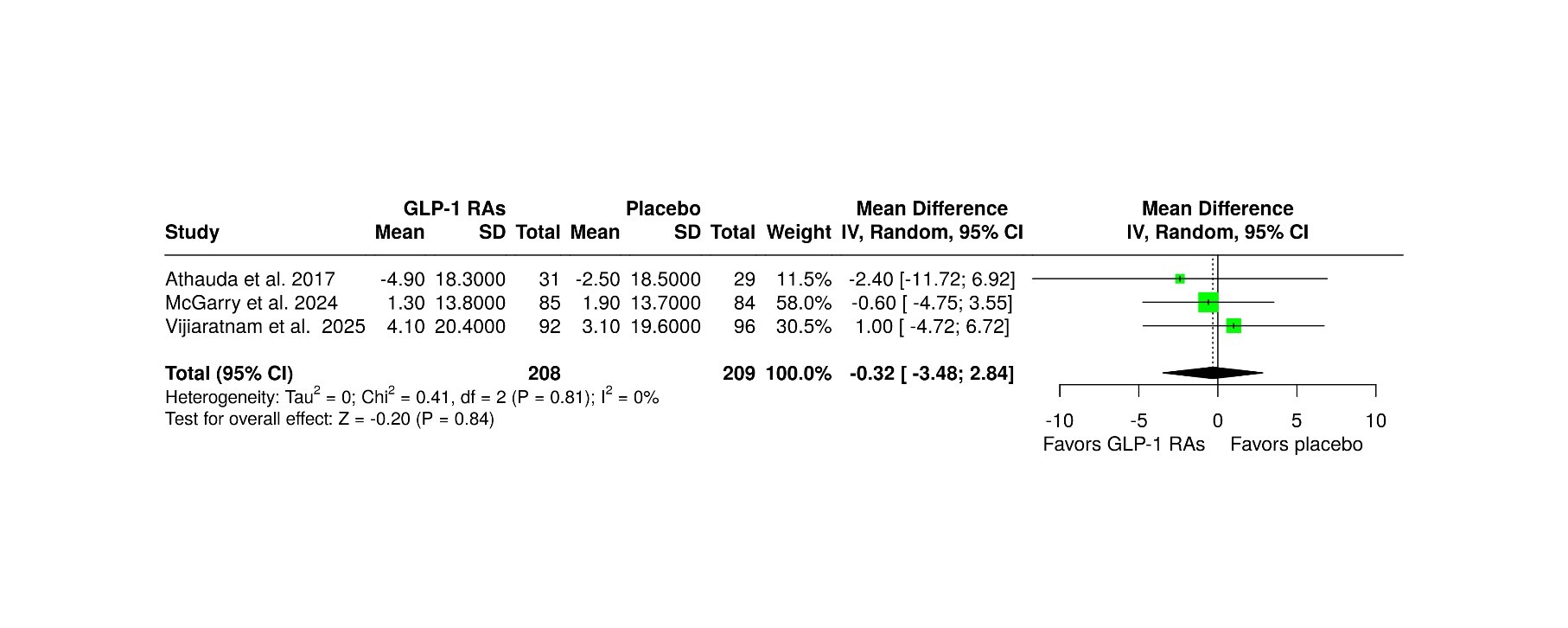
**Figure S32.** Sensitivity analysis using MD (95% CI): Forest plot comparing the changes in the Non-Motor Symptoms Severity Scale at “ON” state in patients with Parkinson’s disease treated with GLP-1 RAs versus placebo.


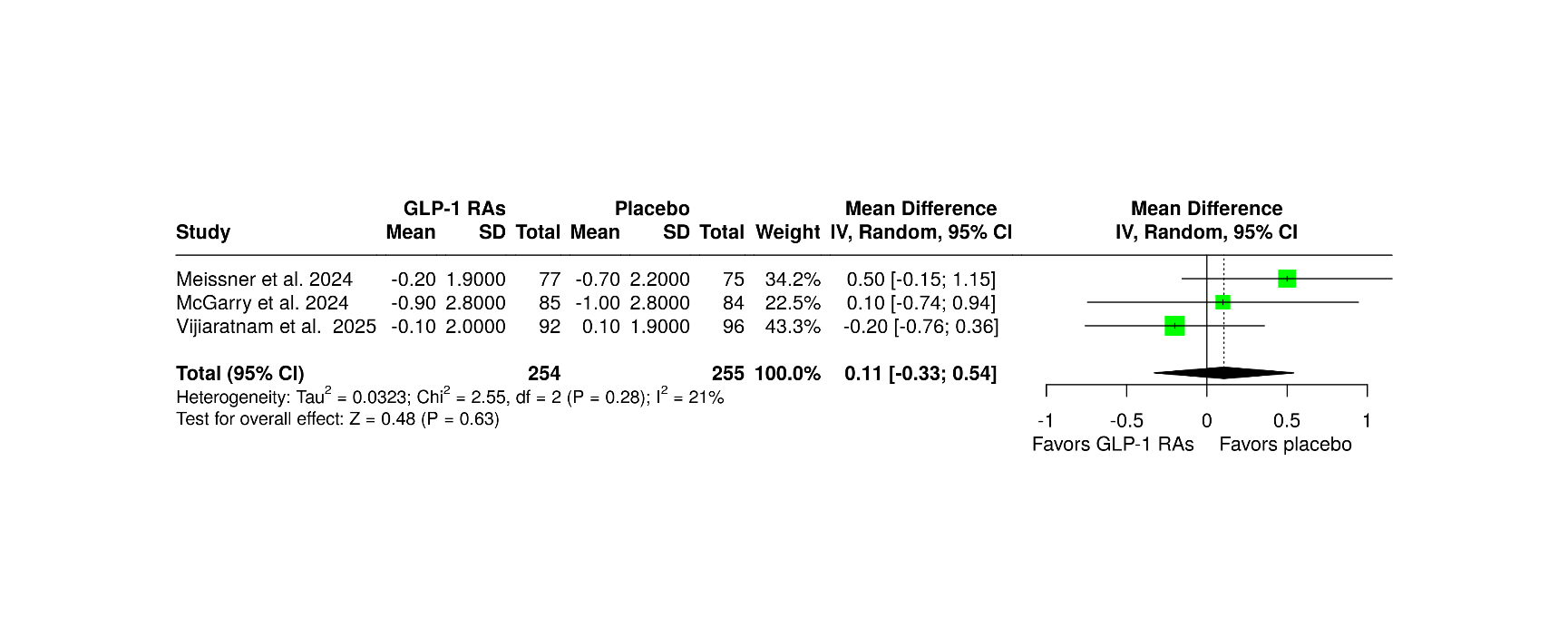
**Figure S33.** Sensitivity analysis using MD (95% CI): Forest plot comparing the changes in the MoCA score at “ON” state in patients with Parkinson’s disease treated with GLP-1 RAs versus placebo.


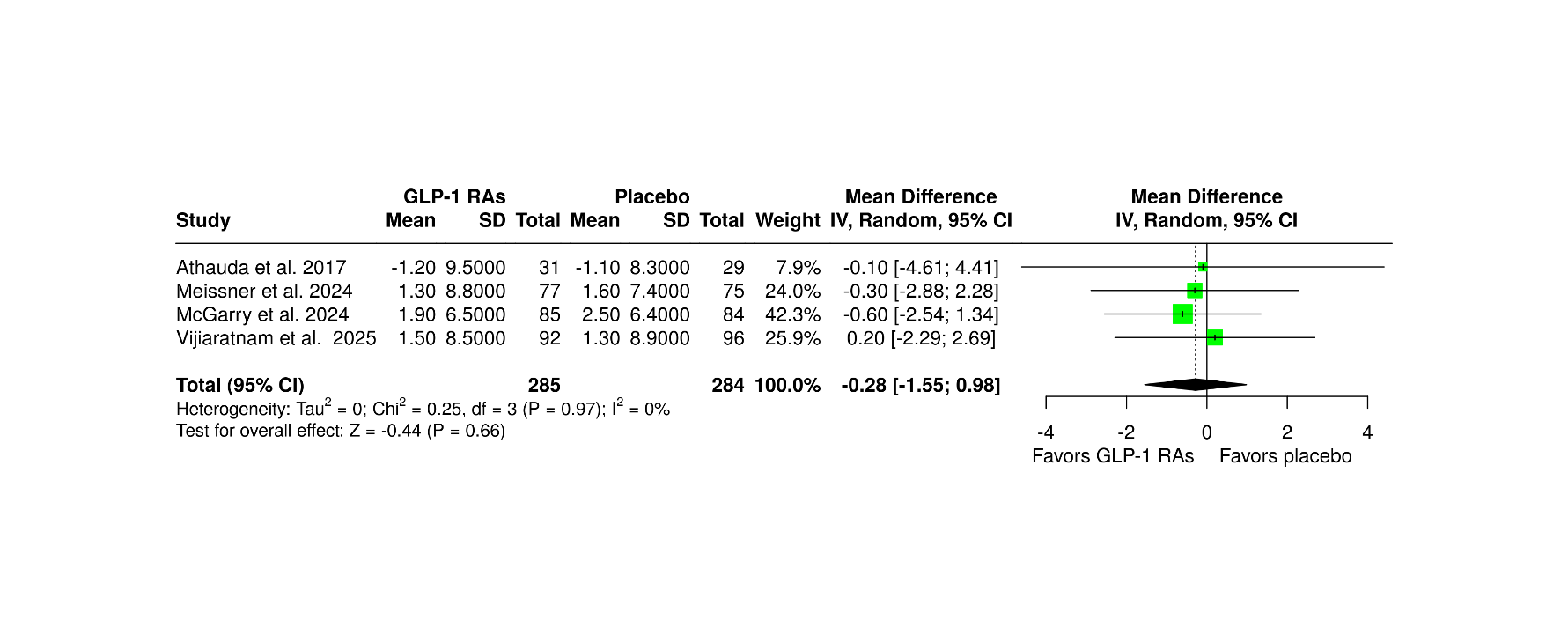
**Figure S34.** Sensitivity analysis using MD (95% CI): Forest plot comparing the changes in the Parkinson’s Disease Questionnaire 39 at “ON” state in patients with Parkinson’s disease treated with GLP-1 RAs versus placebo.

**REFERENCES**

1. Athauda D, Maclagan K, Skene SS, et al. Exenatide once weekly versus placebo in Parkinson's disease: a randomised, double-blind, placebo-controlled trial. Lancet 2017;390(10103):1664-1675.

2. McGarry A, Rosanbalm S, Leinonen M, et al. Safety, tolerability, and efficacy of NLY01 in early untreated Parkinson's disease: a randomised, double-blind, placebo-controlled trial. Lancet Neurol 2024;23(1):37-45.

3. Meissner WG, Remy P, Giordana C, et al. Trial of Lixisenatide in Early Parkinson's Disease. N Engl J Med 2024;390(13):1176-1185.

4. Vijiaratnam N, Girges C, Auld G, et al. Exenatide once a week versus placebo as a potential disease-modifying treatment for people with Parkinson's disease in the UK: a phase 3, multicentre, double-blind, parallel-group, randomised, placebo-controlled trial. Lancet 2025;405(10479):627-636.

5. Sterne JAC, Savović J, Page MJ, et al. RoB 2: a revised tool for assessing risk of bias in randomised trials. Bmj 2019;366:l4898.
